# Supplementary material for: Predictors of genomic differentiation within a hybrid taxon
Source: PLoS Genet. 2022 Feb 11;18(2):e1010027. doi: 10.1371/journal.pgen.1010027 (PMC8870489; doi:10.1371/journal.pgen.1010027)
Supplement: S1 Text — It includes: Table A. Per-island population genomic statistics. Left panel: Mean values of π and within-island genomic differentiation (FST). Middle panel: t-test for pairwise comparison between genome wide within island FST, evaluating a significance difference between genome wide within-island genomic differentiation (FST) across islands. Right panel: Mean values of Tajima’s D per population within each island. Table B. Intercept, slope and confidence intervals of the slope of individual linear regression of within-island genomic differentiation and recombination rate as well as parent-parent differentiation and recombination rate. Table C. Evaluating the effect that the interaction between recombination rate and the type of comparison (parental differentiation (house-Spanish), which is the null model, and within-island differentiation) has on genomic differentiation (FST). Individual linear models per island were run to test if there is a significant interaction between recombination rate and comparison, as expected if the relationship between recombination rate and differentiation differs between parent species and the hybrid Italian sparrow (Fig 1A). Table D. Generalized linear model on within-island FST. Table E. Logistic regressions per island, on the probability of being a local FST outlier within island. Table F. Generalized linear models, separated by island on within-island FST. Table G. Concordance of 1. between-island divergent selection (xp-EHH) and 2. within-island selection (iHS) with genomic differentiation (FST). 3. Correlation between islands of their correspondent within-island selection (iHS) estimates. Table H. Number and percentage of within-island FST outlier loci shared between islands. Chi-squared denote tests for overrepresentation compared to the genome wide average. Table I. Number and percentage of within-island FST outlier loci identical to between-island outliers. Chi-squared denote tests for overrepresentation compared to the genome [file pgen.1010027.s004.docx]

**Predictors of genomic differentiation within a hybrid taxon**

Angélica Cuevas^1*^, Fabrice Eroukhmanoff^1^, Mark Ravinet^1,2^, Glenn-Peter Sætre^1^ and Anna Runemark^3^

**Table A. Per-island population genomic statistics.** Left panel: Mean values of π and *within*-island genomic differentiation (*F*_ST_). Middle panel: t-test for pairwise comparison between within island *F*_ST,_ evaluating a significance difference between genome wide *within*-island genomic differentiation (*F*_ST_) across islands. Right panel: Mean values of Tajima’s D per population within each island.

|  | Mean values | | Comparisons (t-tests) of genome wide within island F_ST_ | | | Tajima’s D | | |
| --- | --- | --- | --- | --- | --- | --- | --- | --- |
|  | π | F_ST_ | t-statistic (*p-value*) | | | Mean values per population (t-test *p-value*) | | |
|  |  |  | **Corsica** | **Crete** | **Sicily** | **Corsica** | **Crete** | **Sicily** |
| **Corsica** | 3.021e-06 | 0.0178 | - |  |  | Muratello | Istro | Cos |
|  |  |  |  |  |  | -0.11 (1.84e-07) | 0.015(0.4882) | -0.25 (< 2.2e-16) |
| **Crete** | 2.863e-06 | 0.0112 | 9.70 | - |  | Pianiccia | Mithimna | Enna |
|  |  |  | (2.2e-16) |  |  | -0.048 (0.016) | -0.011 (0.6257) | -0.185 (< 2.2e-16) |
| **Sicily** | 2.2986e-06 | 0.0134 | 6.24 | 3.47 | - | Tiuccia | Perama | Naxos |
|  |  |  | (4.812e-10) | (0.0005) |  | -0.025 (0.2453) | 0.0087 (0.6983) | -0.172 (< 2.2e-16) |

**Table B. Intercept, slope and confidence intervals of the slope of individual linear regression of *within*-island genomic differentiation and recombination rate as well as parent-parent differentiation and recombination rate.**

| Model: lm(*within*-island *F*_ST_~ Recombination rate) | | | | **95% CI of slope** | |
| --- | --- | --- | --- | --- | --- |
|  | **Intercept** | **Slope** | ***P*-value** | **2.5 %** | **97.5 %** |
| Corsica | 0.018 | -2.5e-04 | 7.83e-01 | -2.03e-03 | 1.5e-03 |
| Crete | 0.0097 | 1.3e-03 | 5.53e-02 | -2.89e-05 | 2.6e-03 |
| Sicily | 0.016 | -2.8e-03 | 5.72e-04 | -4.46e-03 | -1.2e-03 |
| House-Spanish | 0.160 | -0.041 | 1.69e-10 | -0.054 | -0.0295 |

**Table C. Evaluating the effect that the interaction between recombination rate and the type of comparison (parental differentiation (house-Spanish), which is the null model, and within-island differentiation) has on genomic differentiation (*F*_ST_).** Individual linear models per island were run to test if there is a significant interaction between **recombination rate** and **comparison**, as expected if the relationship between recombination rate and differentiation differs between parent species and the hybrid Italian sparrow (Fig 1A).

| lm(*within*-island *F*_ST_~ Recombination Rate * Comparison) | | | | |
| --- | --- | --- | --- | --- |
| Island | Predictor | **Estimate** | **Std. Error** | ***P-value*** |
| Corsica | Recombination rate | -2.50e-04 | 4.4e-03 | 0.955 |
| R^2^ = 0.179 | Comparison | 0.142 | 4.4e-03 | <2.0e-16 |
|  | Recombination rate : Comparison | -0.041 | 6.3e-03 | 4.18e-11 |
| Crete | Recombination rate | 1.28e-03 | 4.58e-03 | 0.77926 |
| R^2^ = 0.200 | Comparison | 0.152 | 4.49e-03 | <2.0e-16 |
|  | Recombination rate : Comparison | -0.042 | 6.47e-03 | 5.51e-11 |
| Sicily | Recombination rate | -2.84e-03 | 4.47e-03 | 0.525 |
| R^2^ = 0.189 | Comparison | 0.144 | 4.40e-03 | <2.0e-16 |
|  | Recombination rate : Comparison | -0.039 | 6.32e-03 | 8.11e-10 |

**Table D. Generalized linear model on *within*-island F_ST_.**

| Model: | | | | |
| --- | --- | --- | --- | --- |
| Within-Island FST = per locus local ancestry proportion (LLAP) + Recombination Rate + Location + island.house FST + island.Spanish FST + house.Spanish FST | | | | |
|  |  |  |  |  |
| **Response variable** | **Predictor** | **Estimate** | **Std. Error** | ***p-value*** |
| Within-island FST | Recombination Rate | -9.66e-04 | 7.84e-04 | 0.22 |
|  | LLAP | 2.09e-04 | 1.31e-03 | 0.87 |
|  | Island vs. House FST | -5.66e-03 | 3.322e-03 | 0.09 |
|  | Island vs. Spanish FST | 3.20e-03 | 2.90e-03 | 0.27 |
|  | House vs. Spanish FST | -3.75e-08 | 3.85e-08 | 0.33 |

| Post-hoc ﻿Estimated marginal (Least-squares) means for predictor variable “island” | | | | | |
| --- | --- | --- | --- | --- | --- |
| **contrast** | **estimate** | **SE** | **t.ratio** | **p.value** |  |
| Corsica - Crete | 0.00608 | 0.000727 | 8.350 | <.0001 |  |
| Corsica - Sicily | 0.00352 | 0.000730 | 4.821 | <.0001 |  |
| Crete - Sicily | -0.00255 | 0.000737 | -3.441 | 0.0015 |  |
| P-value adjustment: tukey | |  |  |  |  |

**Table E. Logistic regressions per island, on the probability of being a local F_ST_ outlier within island.**

| Model: | | | | |
| --- | --- | --- | --- | --- |
| Pr⁡(outlier)= per locus local ancestry proportion (LLAP) + Recombination Rate + island.house FST+ island.Spanish FST + house.Spanish FST | | | | |
|  |  |  |  |  |
| **Response variable** | **Predictor** | **Estimate** | **Std. Error** | ***p-value*** |
| Pr(within-**Corsica** outlier FST) | Recombination Rate | -2.870e-01 | 2.552e-01 | 0.26 |
|  | LLAP | -2.000e-02 | 1.263e-01 | 0.87 |
|  | Island vs. house FST | -1.364e+00 | 1.527e+00 | 0.37 |
|  | Island vs. Spanish FST | 1.267e+00 | 7.405e-01 | 0.09 |
|  | House vs. Spanish FST | -2.419e-05 | 2.349e-05 | 0.30 |
| Pr(within-**Crete** outlier FST) | Recombination Rate | 3.502e-01 | 2.422e-01 | 0.15 |
|  | LLAP | 1.355e-01 | 1.162e-01 | 0.24 |
|  | Island vs. house FST | -1.280e+00 | 1.029e+00 | 0.21 |
|  | Island vs. Spanish FST | 2.476e-01 | 7.027e-01 | 0.73 |
|  | House vs. Spanish FST | 7.579e-06 | 1.007e-05 | 0.45 |
| Pr(within-**Sicily** outlier FST) | Recombination Rate | -4.706e-01 | 2.497e-01 | 0.06 |
|  | LLAP | -5.205e-02 | 9.476e-02 | 0.58 |
|  | Island vs. house FST | -1.441e+00 | 1.078e+00 | 0.18 |
|  | Island vs. Spanish FST | -2.480e-01 | 1.246e+00 | 0.84 |
|  | House vs. Spanish FST | -2.550e-05 | 2.755e-05 | 0.35 |

**Table F. Generalized linear models, separated by island, on within-island F_ST_.**

| Model: | | | | |
| --- | --- | --- | --- | --- |
| Within-Island FST = per locus local ancestry proportion (LLAP) + Recombination Rate + Location + island.house *F*_ST_ + island.Spanish *F*_ST_ + house.Spanish *F*_ST_ | | | | |
|  |  |  |  |  |
| **Response variable** | **Predictor** | **Estimate** | **Std. Error** | ***p-value*** |
| Within-Corsica *F*_ST_ | Recombination Rate | 1.248e-04 | 1.518e-03 | 0.934 |
|  | LLAP | -3.800e-04 | 2.905e-03 | 0.896 |
|  | Island vs House *F*_ST_ | 7.053e-04 | 8.569e-03 | 0.934 |
|  | Island vs. Spanish *F*_ST_ | 1.166e-02 | 5.558e-03 | 0.036* |
|  | House vs. Spanish *F*_ST_ | -1.204e-07 | 7.670e-08 | 0.117 |
| Within-Crete *F*_ST_ | Recombination Rate | 7.032e-04 | 1.186e-03 | 0.553 |
|  | LLAP | 2.119e-03 | 2.013e-03 | 0.293 |
|  | Island vs. House *F*_ST_ | -5.490e-03 | 4.549e-03 | 0.228 |
|  | Island vs. Spanish *F*_ST_ | 1.199e-04 | 3.571e-03 | 0.973 |
|  | House vs. Spanish *F*_ST_ | 1.101e-08 | 5.628e-08 | 0.845 |
| Within-Sicily *F*_ST_ | Recombination Rate | -3.475e-03 | 1.336e-03 | 9.3e-03** |
|  | LLAP | -8.502e-04 | 1.975e-03 | 0.667 |
|  | Island vs. House *F*_ST_ | -8.852e-03 | 5.139e-03 | 0.085 |
|  | Island vs. Spanish *F*_ST_ | -4.204e-03 | 7.033e-03 | 0.550 |
|  | House vs. Spanish *F*_ST_ | -1.191e-08 | 6.615e-08 | 0.857 |

**Table G. Concordance of 1. *between*-island divergent selection (xp-EHH and 2. *within*-island selection (iHS) with genomic differentiation (*F*_ST_**)**. 3.** Correlation between islands of their correspondent *within*-island selection (iHS) estimates.

| **1. *Between*-island *F*_ST_ ~ xp-EHH** | | |  |  | |
| --- | --- | --- | --- | --- | --- |
|  | **t-estimate** | **df** | **Correlation** | **Multiple R^2^** | ***p-value*** |
| Corsica vs. Crete | -1.967 | 2143 | -0.042 | 0.0018 | 0.148 |
| Corsica vs. Sicily | -2.8314 | 2140 | -0.061 | 0.0037 | 0.014* |
| Crete vs. Sicily | -2.7373 | 2113 | -0.059 | 0.0035 | 0.019* |
|  |  |  |  |  |  |
| **2. *Within*-island *F*_ST_ ~ iHS** | |  |  |  |  |
|  | **t-estimate** | **df** | **Correlation** | **Multiple R^2^** | ***p-value*** |
| Corsica | 0.14606 | 1378 | 0.0039 | 1.55e-5 | 0.884 |
| Crete | 0.41902 | 1292 | 0.0117 | 1.36e-4 | 0.675 |
| Sicily | 2.5594 | 1255 | 0.0721 | 0.0052 | 0.0106* |
|  |  |  |  |  |  |
| **3. Pair wise Pearson correlations of iHS** | | |  |  |  |
|  | **t-estimate** | **df** | **Correlation** | **Multiple R^2^** | ***p-value*** |
| Corsica vs. Crete | 9.286 | 822 | 0.308 | 0.095 | 6.6e-16 |
| Corsica vs. Sicily | 13.135 | 822 | 0.417 | 0.174 | 6.6e-16 |
| Crete vs. Sicily | 10.214 | 822 | 0.336 | 0.113 | 6.6e-16 |

Note: Bonferroni corrections of p-values were applied to control for multiple testing.

**Table H. Number and percentage of *within*-island *F*_ST_ outlier loci shared between islands.** Chi-squared denote tests for overrepresentation compared to the genome wide average.

|  |  | **Within-islands windowed-*F*_ST_ outliers** | | | | | |
| --- | --- | --- | --- | --- | --- | --- | --- |
|  |  | Corsica N= 378 | | Crete N= 402 | | Sicily N= 423 | |
|  |  | Number of shared loci  (Chi-squared) | % | Number of shared loci  (Chi-squared) | % | Number of shared loci  (Chi-squared) | % |
| **Within-islands windowed-*F*_ST_ outliers** | Corsica | - | - | 35 | 8.7% | 35 | 8.3% |
|  | Crete | 35  (7.18, *P:* 0.007**) | 9.3% | - | - | 23 | 5.4% |
|  | Sicily | 35  (6.80, *P:* 0.009**) | 9.3% | 23  (0.09, *P*: 0.767) | 5.7% | - | - |

**Table I. Number and percentage of *within*-island *F*_ST_ outlier loci identical to *between*-island *F*_ST_ outliers.** Chi-squared denote tests for overrepresentation compared to the genome wide average.

|  |  | **Within-islands windowed-*F*_ST_ outliers** | | | | | |
| --- | --- | --- | --- | --- | --- | --- | --- |
|  |  | Corsica N= 378 | | Crete N= 402 | | Sicily N= 423 | |
|  |  | shared loci | % | shared loci | % | shared loci | % |
|  |  | (Chi-squared) |  | (Chi-squared) |  | (Chi-squared) |  |
| **Between-islands windowed-*F*_ST_ outliers** | Corsica vs. Crete | 20 | 5.3% | 25 | 6.2% | 11 | 2.6% |
|  |  |  |  | (0.922, *P*: 0.34) |  |  |  |
|  | Corsica vs. Sicily | 37 | 9.8% | 20 | 5.0% | 21 | 5.0% |
|  |  | (15.525, *P*: 8.1e-05^***^) |  | (0.091, *P*: 0.76) |  |  |  |
|  | Crete vs. Sicily | 28 | 7.4% | 41 | 10.2% | 26 | 6.1% |
|  |  | (4.085, *P*: 0.04^*^) |  | (21.129, *P*: 4.3e-06^***^) |  | (1.023, *P*: 0.3) |  |

**Table J**. **Parallel vs. background selection.** *F*_ST_ comparisons between within-island subpopulations across all islands. *P-*value, correlations estimates and t-estimates are corrected for multiple testing by resampling and taking mean estimates after 100 iterations of correlations.

| **Comparison** | **t-estimate** | **df** | **correlation** | **Corrected p-value** |
| --- | --- | --- | --- | --- |
| Sic_Cos.En vs. Cor_Mu.Ti | 1.681115 | 1498 | 0.04 | 0.13 |
| Cor_Pi.Ti vs. Sic_Cos.En | 1.681115 | 1498 | 0.04 | 0.13 |
| Cor_Pi.Ti vs. Sic_Cos.Na | 1.529509 | 1498 | 0.04 | 0.17 |
| Cor_Pi.Ti vs. Sic_En.Na | 2.395912 | 1498 | 0.06 | 0.05 |
| Cre_Mi.Pe vs. Sic_Cos.En | 2.110884 | 1498 | 0.05 | 0.08 |
| Cre_Is.Pe vs. Sic_En.Na | 1.820645 | 1498 | 0.05 | 0.11 |

**Table K**. **Linear model of recombination rate and minor-parent ancestry across islands.** The models are performed using values of Log10 of recombination rate as a predictor of local ancestry (LLAP) and dividing these in quartile bins to group the recombination rate values and facilitate interpretation.

| **Linear model**  Using quartile bins of Log10 of recombination rate | **Estimate** | **Std. Error** | **Adjusted R2** | ***p-value*** | **Island** |
| --- | --- | --- | --- | --- | --- |
|  | 0.0012136 | 0.0001036 | 0.0005732 | < 2.2e-16 | Corsica |
|  | -0.0005305 | 0.0001072 | 7.969e-05 | 7.47e-07 | Crete |
|  | -0.0088202 | 0.000148 | 0.008203 | < 2.2e-16 | Sicily |

**Table L. Different cut-offs for the Analysis of Molecular Variance (AMOVA) across islands and populations within islands.** Several cut-offs for missing-ness per loci were used: 5% (see Table 1), **1.** 10% and **2.** 20%, but the results from the AMOVA did not change substantially.

| **1.** Analysis of Molecular Variance | | | | | Randomization by Permutation | |
| --- | --- | --- | --- | --- | --- | --- |
| AMOVA | | | | | Monte Carlo test | |
| **Variance partitioning** | **Df** | **Sum Sq** | **Sigma** | **% of covariance** | **Std. Observed** | ***P-value*** |
| Among islands | 2 | 778.79 | 2.21 | 4.83 | 4.33 | 0.001 |
|  |  |  |  |  |  |  |
| Between populations within island | 6 | 365.37 | 0.42 | 0.91 | 9.42 | 0.001 |
|  |  |  |  |  |  |  |
| Between individuals within populations | 213 | 8654.88 | -2.54 | -5.54 | -4.17 | 1.000 |
|  |  |  |  |  |  |  |
| Within individuals | 222 | 10148.04 | 45.71 | 99.80 | 1.26 | 0.897 |
|  |  |  |  |  |  |  |

| **2.** Analysis of Molecular Variance | | | | | Randomization by Permutation | |
| --- | --- | --- | --- | --- | --- | --- |
| AMOVA | | | | | Monte Carlo test | |
| **Variance partitioning** | **Df** | **Sum Sq** | **Sigma** | **% of covariance** | **Std. Observed** | ***P-value*** |
| Among islands | 2 | 778.79 | 2.21 | 4.84 | 4.40 | 0.001 |
|  |  |  |  |  |  |  |
| Between populations within island | 6 | 365.37 | 0.42 | 0.91 | 9.41 | 0.001 |
|  |  |  |  |  |  |  |
| Between individuals within populations | 213 | 8654.87 | -2.54 | -5.54 | -4.31 | 1.000 |
|  |  |  |  |  |  |  |
| Within individuals | 222 | 10148.04 | 45.71 | 99.79 | 1.26 | 0.901 |
|  |  |  |  |  |  |  |

**
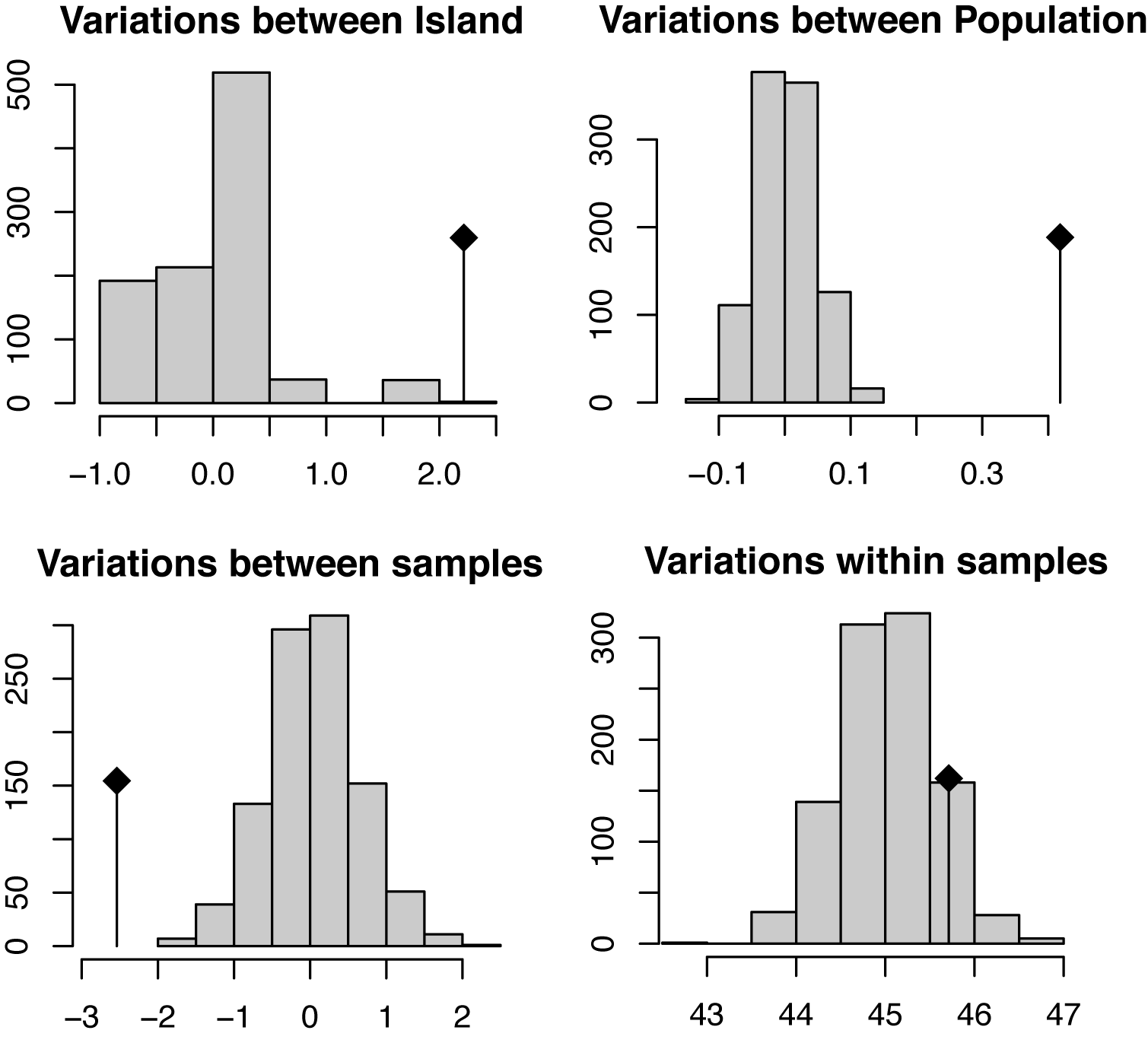
**

**Fig A. AMOVA significance - Randomization via permutation.** Monte Carlo test with 1000 permutations implemented in the randtest() function from the ADE4 R-package to evaluate significance. Black line denotes the observed values of Sigma (Variance in each hierarchical level).


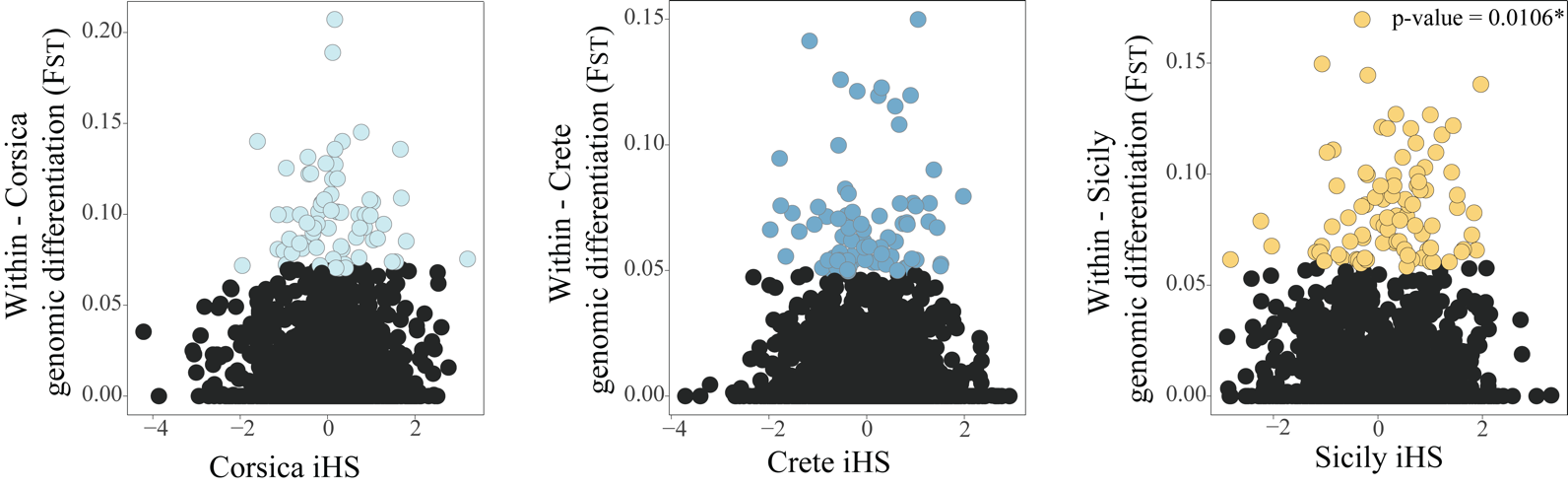


**Fig B. Concordance of patterns of selection and genomic differentiation.** Correlations of the integrated haplotype homozygosity score (iHS) and genomic differentiation (within-island FST). 1% FST outliers are indicated in coloured dots in contrast to the non-outlier loci, in black.

**
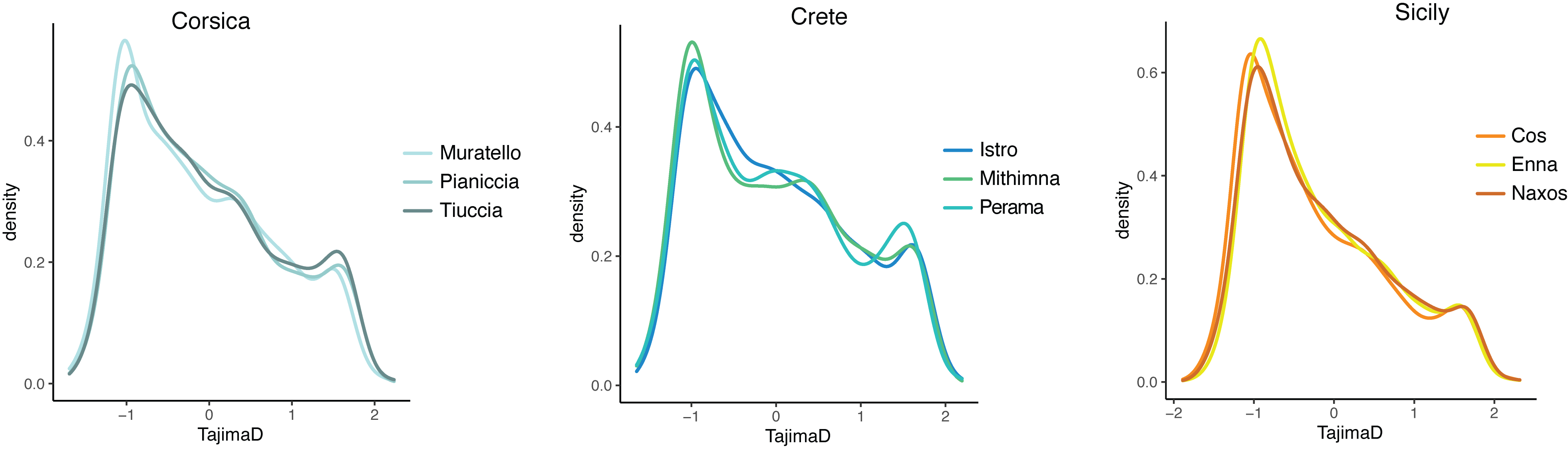
**

**Fig C. Distribution of Tajima’s D per population in each island.**


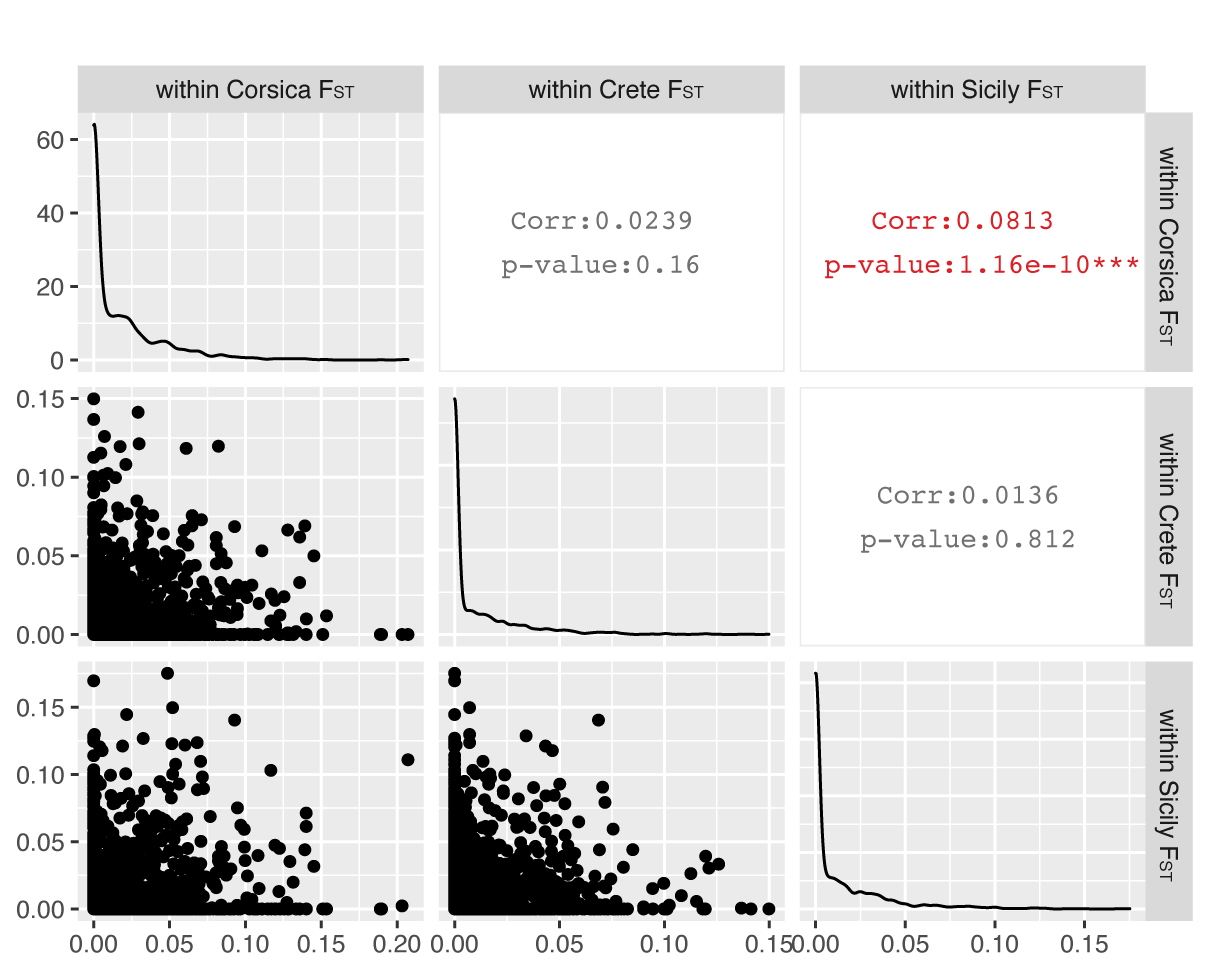


**Fig D. Correlation of within-islands differentiation across the three Mediterranean islands.** Bonferroni corrections of the p-values are reported.

**
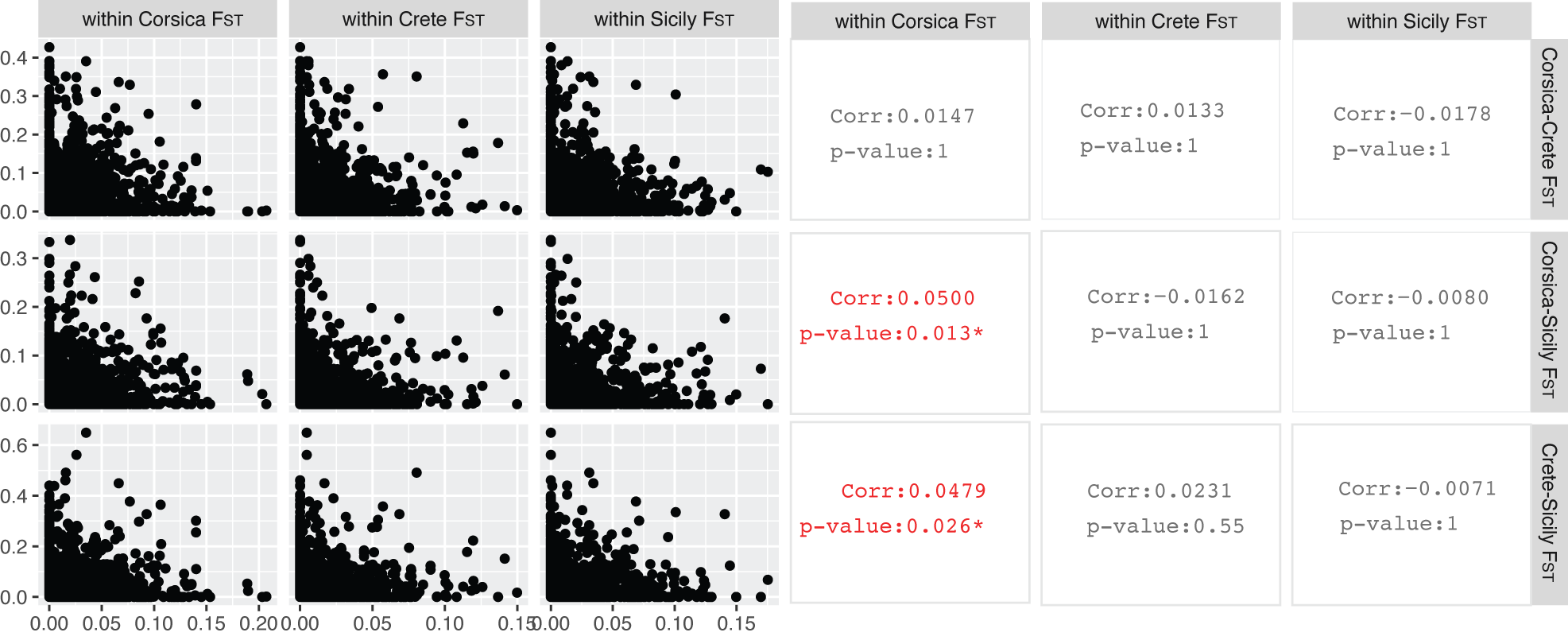
**

**Fig E. Correlation of within-islands differentiation vs. between-islands divergence.** Adjusted p-values after resampling and Bonferroni corrections.


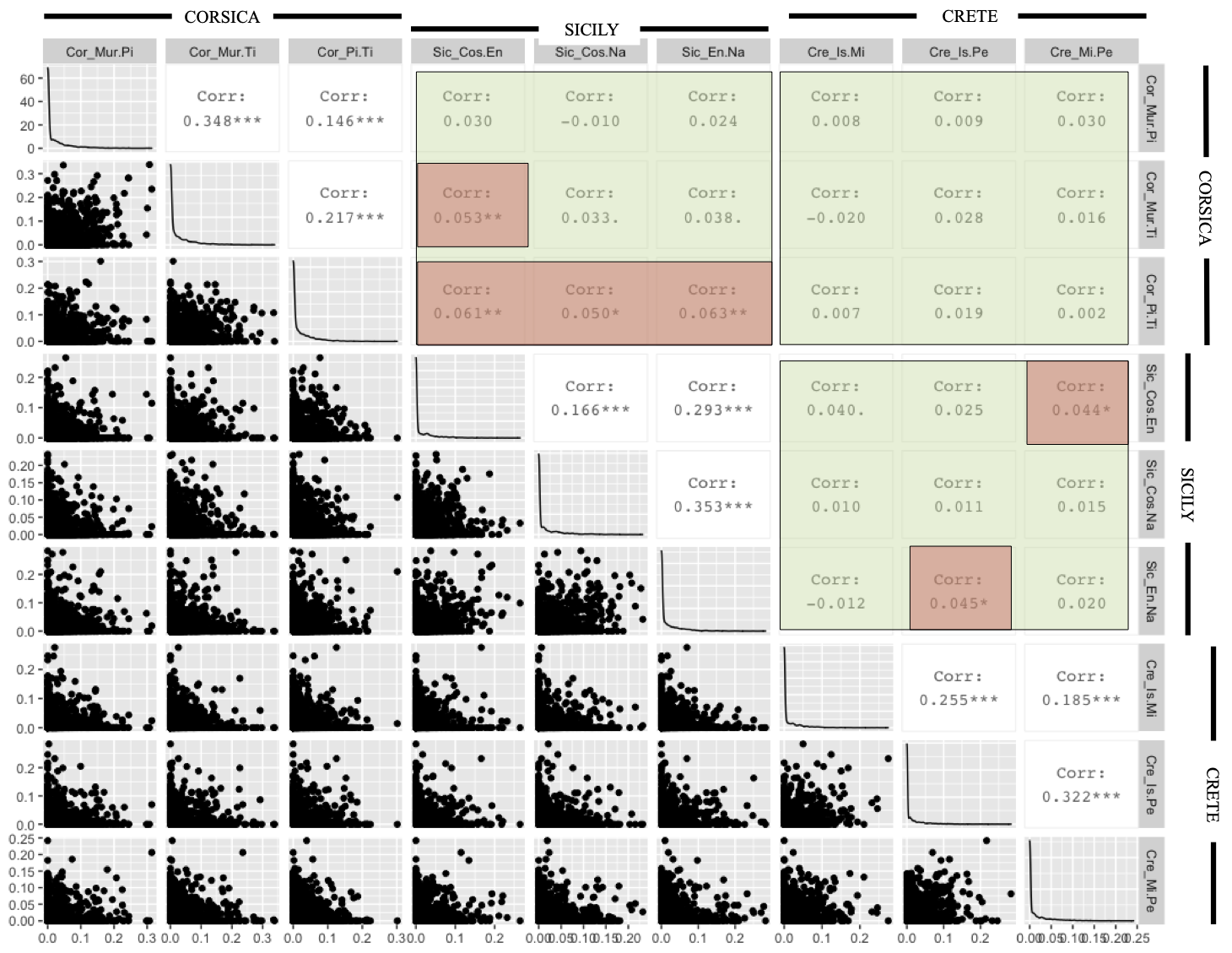


**Fig F. Parallelism of *within*-island pairwise FST**. Pairwise *F*_ST_ correlations between populations within island “A” to pairwise *F*_ST_ estimates of populations within island “B” highlighted in green. Significant correlations before correction for multiple testing highlighted in red. Abbreviations of the comparisons are as follow: CORSICA populations: Muratello (Mur), Pianiccia (Pi), Tiuccia (Pi). CRETE populations: Istro (Is), Mithimna (Mi), Perama (Pe). SICILY populations: Cos (Co), Enna (En), Naxos (Na). Thus pair-wise *F*_ST_ between Muratello vs. Pianiccia is abbreviated as “*Cor_Mur.Ti*”. Similarly, pair-wise *F*_ST_ between Enna vs. Naxos is abbreviated as “*Sic_En.Na*”. Estimate values are corrected for multiple testing using a resampling approach (Table J).

**
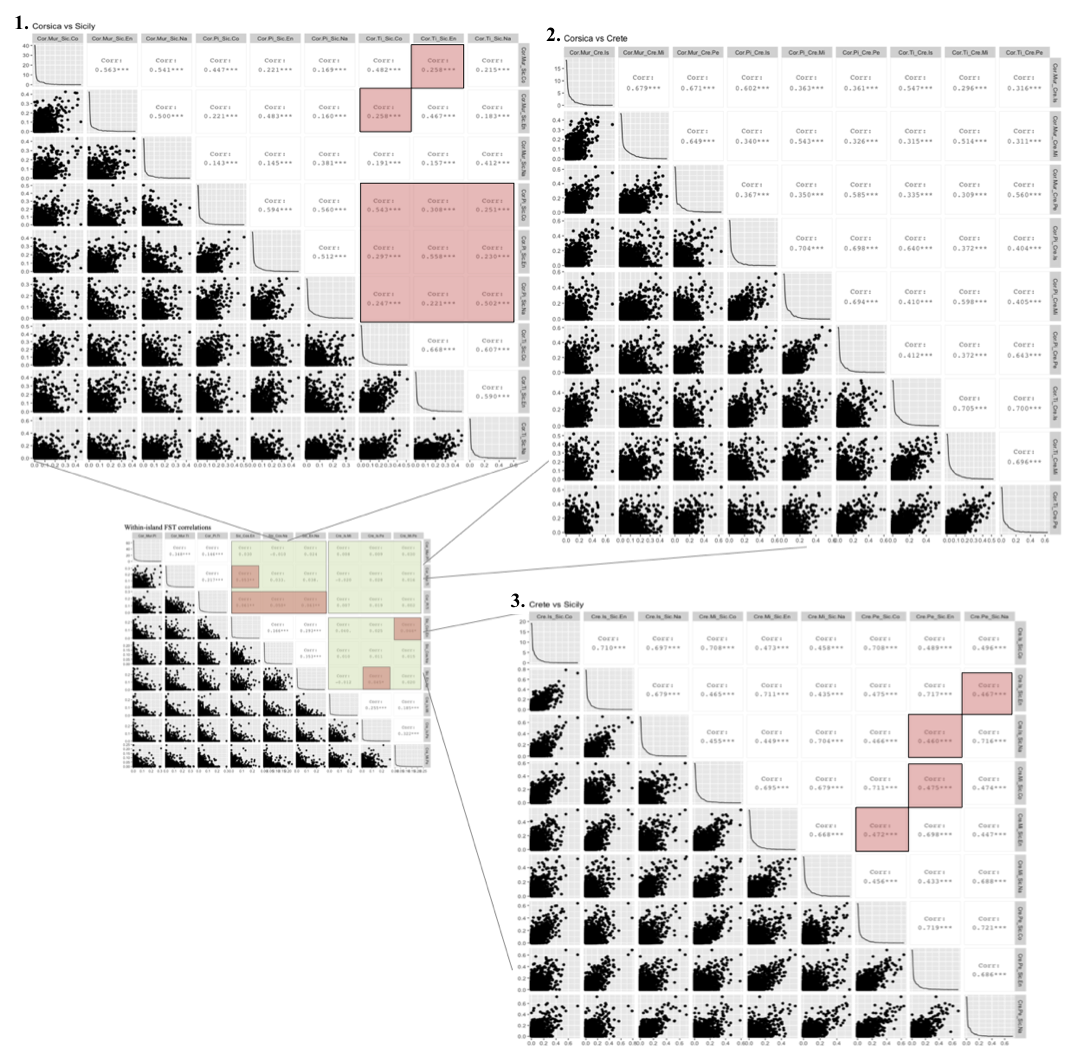
**

**Fig G. Parallelism of *between*-island pairwise *F*_ST_ across all subpopulations.** Correlations of pairwise-*F*_ST_ between subpopulation a (from island “A”) and b (from island “B”) and its contrast pairwise-*F*_ST_ between subpopulation a’ (from island “A”) and b’ (from island “B”). **1.** Correlations between Corsican vs. Sicilian subpopulations. **2.** Corsican vs. Cretan subpopulations and **3.** Sicilian vs. Cretan subpopulations. Populations’ name of each island are presented in Fig F. Abbreviations of the comparisons are as follow: As an example, pair-wise *F*_ST_ between Muratello (from Corsica) vs. Enna (from Sicily) is abbreviated as “*Cor.Mur_Sic.En*”. Similarly, pair-wise *F*_ST_ between Perama (from Crete) vs. Naxos (from Sicily) is abbreviated as “*Cre.Pe_Sic.Na*”.


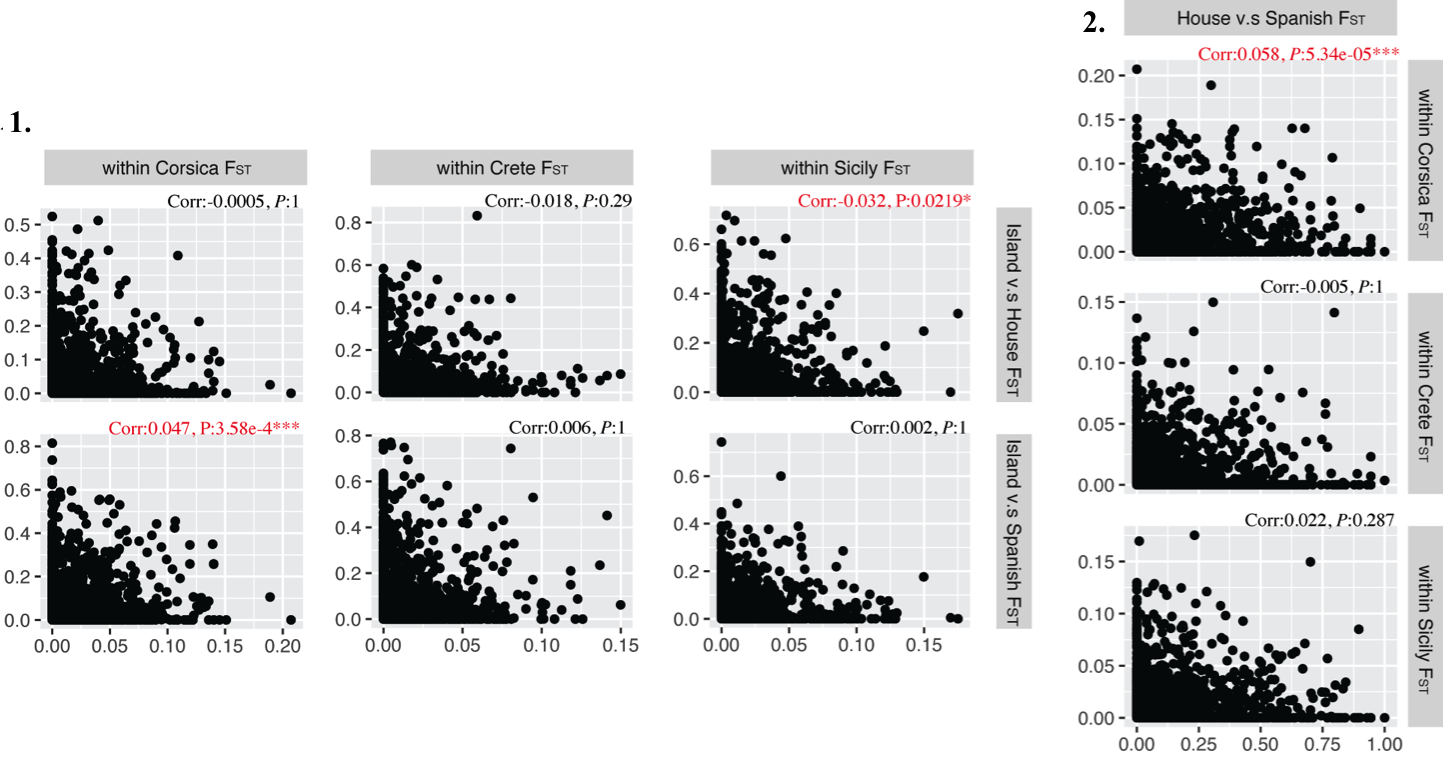


**Fig H. Correlation of within-islands differentiation and the parental species.** Adjusted p-values after Bonferroni corrections.


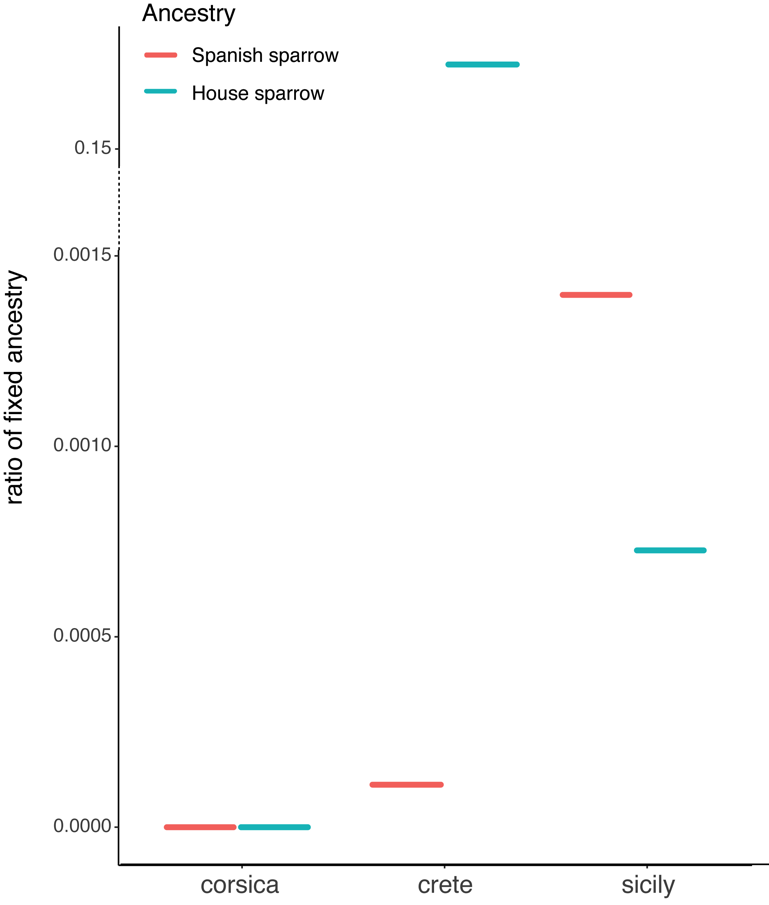


**Fig I.** **Fixation rate of parentally differentiated fixed sites across the islands Italian sparrow populations.** Fixation rate is presented individually by ancestry. Continuity of the y-axis is broken (dashed line) to minimize the size of the figure in order to include the extreme values of the distribution.

**
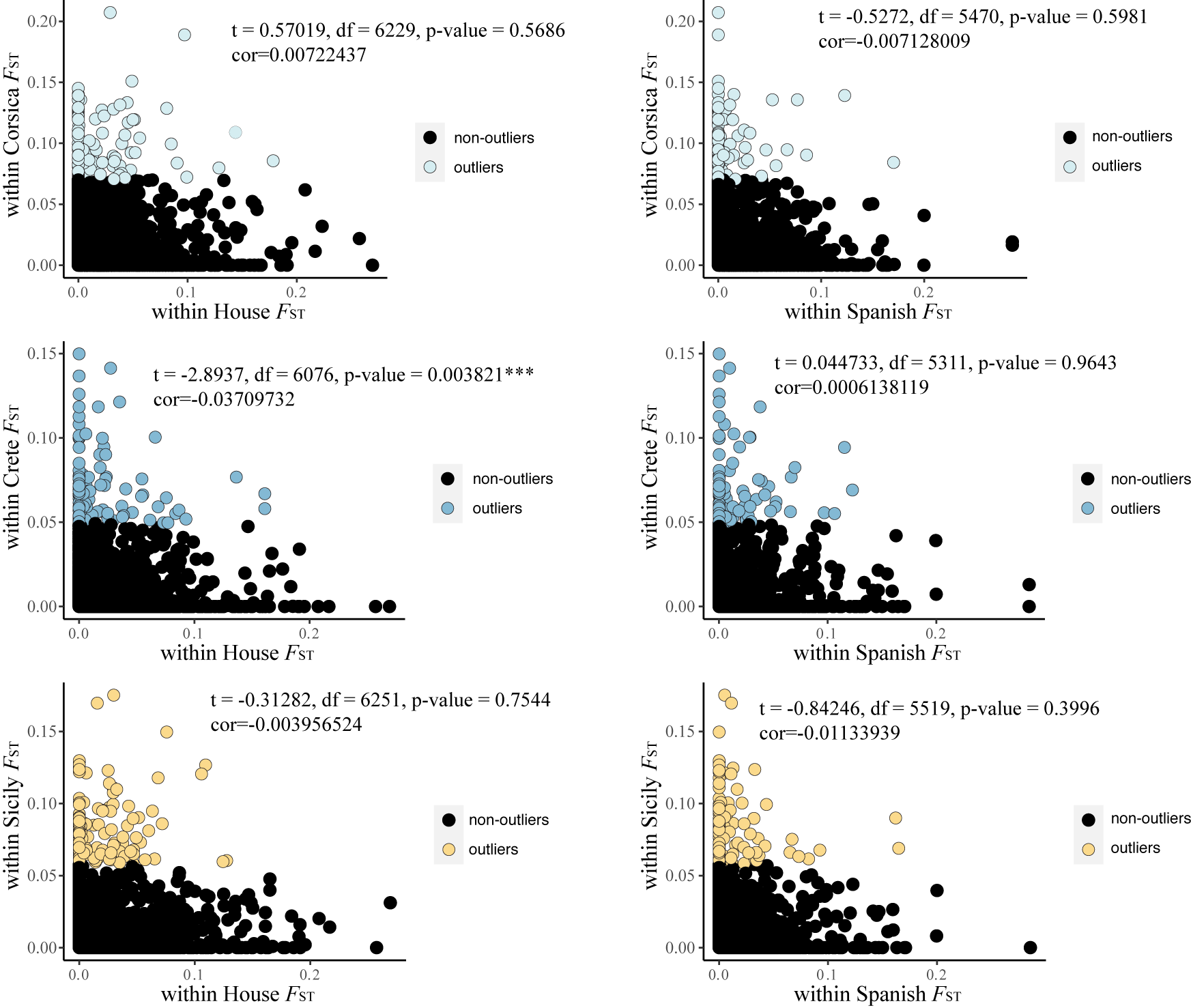
**

**Fig J. Correlations of within-island differentiation and within-parent differentiation (within-house or and within-Spanish sparrow)**. 1% *F*_ST_ outliers are indicated in coloured dots in contrast to the non-outlier loci, in black.

**
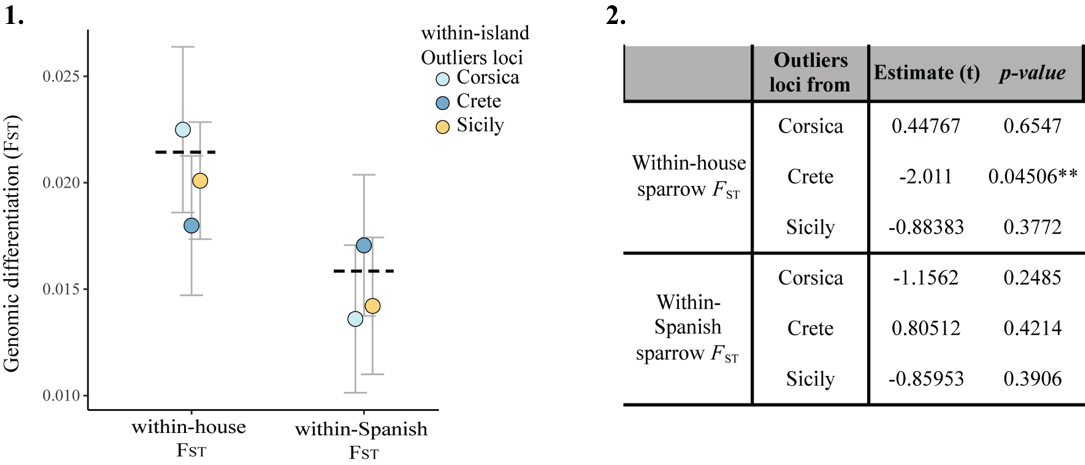
**

**Fig K. 1.** Intraspecific genomic differentiation in the parental species for the within-island F_ST_ outlier loci. Dash lines represent the within-parent F_ST_ global mean. **2.** t-tests evaluating whether within-island outlier loci present higher/lower values than expected by chance in the within-parent differentiation.


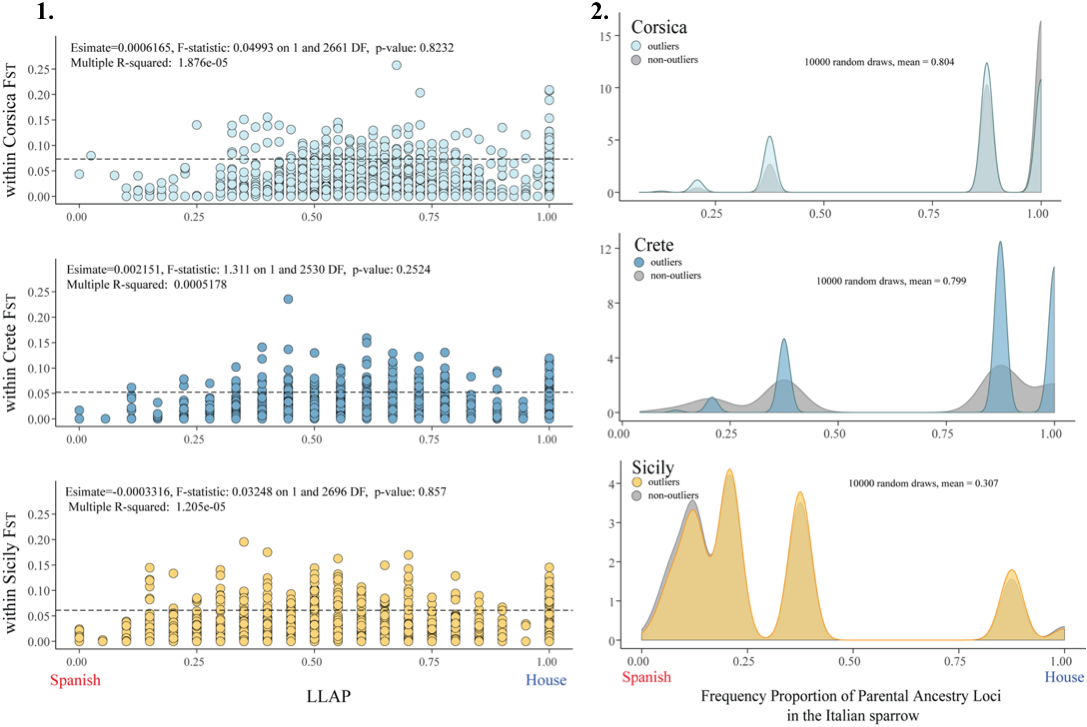


**Fig L.** **1.** Relation between within-island F_ST_ and per locus local ancestry proportion (LLAP). Results of linear regression reported. Dashed lines depict the 1% outliers threshold. **2.** Frequency proportion of outlier loci found in regions of mainly house ancestry (0.65< LLAP) and mainly Spanish ancestry (LLAP <0.35) (minor-major parental ancestry). Distribution of 10.000 random resampling draws of 8 outlier loci.

**
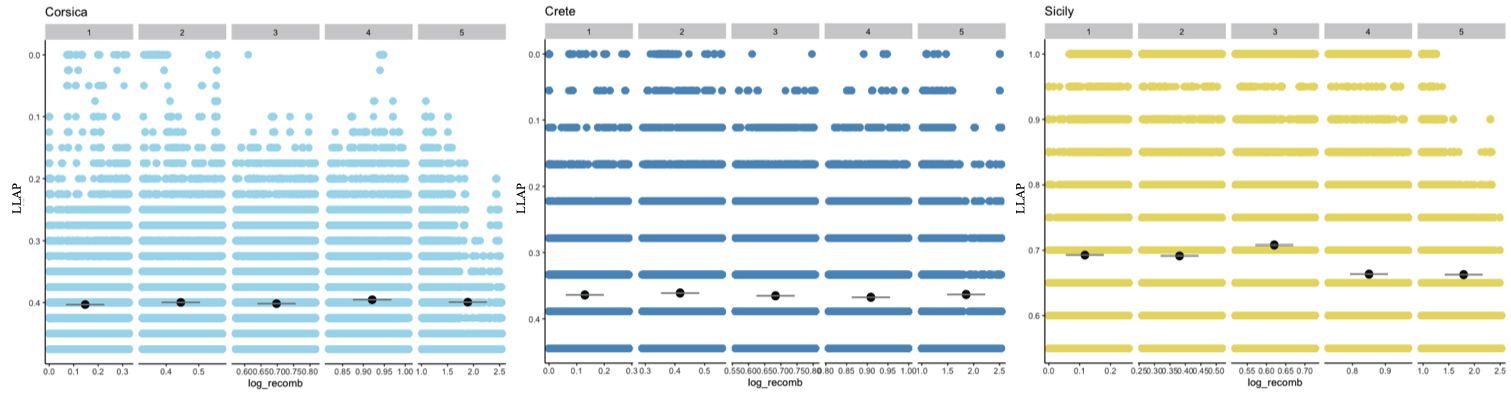
**

**Fig M.** **Recombination rate v.s proportion of minor-ancestry** (using LLAP, where values of 1 = 100% house ancestry and 0=100% Spanish ancestry). Recombination rate is presented in quartiles using whole genome resequencing data retrieved from Ravinet et al (2018), Elgvin et al (2017) and Runemark et al (2018a). Mean and confident intervals of LLAP are shown per recombination rate quantile. Minor ancestors are as follow: Corsica: minor-ancestry from the Spanish sparrow (LAAP= 0 to 0.5), n=237.523 SNPs. Crete: minor-ancestry Spanish sparrow (LAAP= 0 to 0.5), n=294.749SNPs and Sicily: minor-ancestry the house sparrow (LAAP= 0.5 to 1), n=424.739SNPs.
