# Supplementary material for: Predictors of genomic differentiation within a hybrid taxon
Source: PLoS Genet. 2022 Feb 11;18(2):e1010027. doi: 10.1371/journal.pgen.1010027 (PMC8870489; doi:10.1371/journal.pgen.1010027)
Supplement: Attachment — Submitted filename: Response.Letter_Cuevas.etal.2021_PLoSGenetics.docx [file pgen.1010027.s005.docx]

**October 1^st^ 2021**

Dear Editor,

We would like the editors to consider our manuscript entitled “Predictors of genomic differentiation within a hybrid taxon”. This is a substantially revised version of the manuscript PGENETICS-D-21-00438. We have made extensive changes to the manuscript following the recommendations of the editor and referees, including:

- Clarified the expectations for the patterns of differentiation under different scenarios
- Provided novel analyses to disentangle background selection from adaptive parallel selection
- Clarified to what extent the hybrid genomes are stabilized, provided analyses on fixation rate as a proxy for genome stabilization and discussed how this influences our findings
- Included a list of relevant genes in or adjacent (within the limit of linkage decay) to the shared highly differentiated genomic regions across islands

We have carefully revised the manuscript, taking all suggestions by the reviewers into consideration. Please find a detailed response to the editor and reviewer’s comments below. We hope the editor will find this revised version suitable for publication in PLoS genetics. We anticipate this research to provide crucial new insights into the evolutionary processes molding hybrid genomes and its implication for the potential of hybridization to contribute new diversity.

Sincerely yours,

**Angélica Cuevas (corresponding author)**

[a.m.c.pulido@ibv.uio.no](mailto:a.m.c.pulido@ibv.uio.no), [angelica.cuevas@evobio.eu](mailto:angelica.cuevas@evobio.eu)

Centre for Ecological and Evolutionary Synthesis

University of Oslo, Norway

<https://www.mn.uio.no/cees/english/people/phd/angelimc/>

---------------------------------------------------------------------------------------------------------------------

**Comments to the Authors:**

This manuscript has been reviewed carefully by three referees, each of whom notes their interest in this comparison among the multiple products of hybridization. The reviews provide many suggestions for improvement of the presentation in the manuscript, with explicit suggestions for changes and questions about specifics. Additionally, one review raises an important point about the distinction between the process of sorting of ancestry blocks in genome stabilization and additional evolutionary processes that operate thereafter or simultaneously on genetic variants within ancestry blocks. At present, this complexity associated with hybrid speciation is not incorporated.

**RESPONSE:** We are very happy that the referees find our study interesting, and we appreciate their constructive comments to which we have answered in detail below. We have now revised the analyses, added further analyses and reworked the interpretation along the lines suggested by the reviewers to make this a solid manuscript fulfilling the potential to address the objectives. In this revised version, we have modified the manuscript to address how the process of hybrid genome stabilization and later evolutionary processes could lead to complex patterns of differentiation in a hybrid taxon. Specifically, we have now compared fixation rate of parental differentiated loci among islands, we are using this metric as a proxy for genome stabilization. While stabilization may not necessarily imply fixation, rate of fixation of parental allele occurring to reduce incompatibilities could reflect on the level of genomic stabilization (see the response to reviewer #1 point 2). These results are now included in the manuscript (Lines 659 - 673; Lines 857 - 868; Figure S9).

We have also referred to previous findings by Trier et al., (2014) *PLoS Genetics,* on the fixation of house mitonuclear and DNA-repair loci and Runemark et al., (2018) *Nature Ecol Evol*, on ancestry tract length distribution and ancestry tracts between islands. These data can further inform us on the level of genome stabilization occurring on these islands populations.

Overall the reviews are very positive about the manuscript's objectives but raise questions about the analyses that were performed and the interpretation. To the concerns about analysis I will add the concern that concordance of Fst between sampling groups could arise because of unusually intermediate allele frequency at these loci in the ancestors, rather than a shared history of selection, given that much of the variation in Fst is driven by the marginal allele frequency of the minor allele at bivariate sites (see 10.1534/genetics.112.144758).

I encourage the authors to consider these suggestions and encouragement for improvement of the manuscript, while recognizing that addressing some of the points would likely require additional analyses.

**RESPONSE:** We thank the reviewers and the editor for their valuable comments and have modified the manuscript accordingly.

We have addressed the possibility that intermediate allele frequency in ancestors could explain the concordance of F_ST_ between sampling groups through testing for the relationship between **the most frequent allele in the total population** **(M)** and FST, where FST takes its maximum values around M=0.5 in a biallelic case (see below Fig1 of Jakobsson et al., 2013), as reported by the reference the Editor suggested, Jakobsson et al., (2013).

**Figure 1 from Jakobsson et al., 2013.**

Jakobsson et al., (2013) define **M** as the average of the highest allele frequencies in both populations. M = σ_1_/2, where σ_1_ is the sum of frequencies of allele 1 across the 2 populations (both parental populations in our case). σ_1_ = *p_11_ + p_21_,* where *p_11_* is the frequency of allele 1 in population 1 and *p_21_* is the frequency of allele 1 in population 2 (see table 1)*.* Values of M=0.5 can be achieved by having intermediate allele frequencies in both parents: *p_11_*=0.5, *p_21_*=0.5; as well as non-intermediate allele frequencies *p_11_*=0.1, *p_21_*=0.9.

Using RAD data from the reference parental populations (2804 SNPs) we compared values of M across the genome and the shared regions of high within-island differentiation.

Overall, we do not find a higher proportion of intermediate values of M (M=0.5), as would be expected if, as the editor suggests, shared patterns of differentiation are an artefact of the allele frequency of the ancestral populations (Table R1).

| **Whole genome vs.**  **shared outlier loci** | Welch Two Sample t-test (assuming non-equal variances) | | | Kolmogorov-Smirnov test of equal distribution | |
| --- | --- | --- | --- | --- | --- |
|  | **t** | **df** | ***P*** | **D** | ***P*** |
| corsica-crete | 1.7197 | 33.665 | 0.0946 | 0.27403 | 0.01288 |
| corsica-sicily | 3.873 | 34.782 | 0.0005* | 0.42562 | 7.27e-06* |
| crete-sicily | 0.30618 | 22.259 | 0.7623 | 0.27667 | 0.06086 |

Table R1. Mean and distribution comparisons of the most frequent allele (M) across the whole genome and the shared regions of high *within*-island differentiation.

Shared *within*-island outlier loci between Corsica and Sicily vary in their mean and distribution from the genome-wide pattern, however, they do not show higher proportion of values of M=0.5 (Fig. R1).

**Figure R1.** Distribution of M, estimated from the parental population, across the shared within-island outlier loci and whole genome values.

It is also important to point out that as shown by Fig1 of Jakobsson et al., (2013) (see above) the implication of intermediate values of M in a biallelic case (M = 0.5) is that the lower and upper bounds for FST are actually between 0 and 1. This suggests that in the case of an excess of intermediate values of M, values of F are less constrained and thus we would actually expect less concordance among populations.

**Reviewer's Responses to Questions**

**Reviewer #1:**

This manuscript describes patterns of genetic differentiation among populations of Italian sparrows. Past work has shown that Italian sparrows likely evolved following hybridization between house and Spanish sparrows. In addition to Italian sparrows in mainland Europe, Italian sparrow populations exist on several islands in the Mediterranean. These island populations are the focus of this manuscript. Specifically, this manuscript documents patterns of genetic differentiation among Italian sparrow populations within islands and between populations on different islands. A suite of analyses are then used to determine whether and to what extent patterns of genetic differentiation can be explained by recombination rate variation, genetic differentiation between the parental species, etc.

I generally liked the overall aims and focus of the paper. In particular, I think comparing multiple outcomes of the genome stabilization process provides insights into how selection and recombination (and drift) shape hybrid genome composition. However, I think more clarity and additional analyses are needed before robust conclusions can drawn from the results in this paper. My biggest concerns are as follows:

1. During genome stabilization, ancestry blocks (segments) fix by drift and selection (with sizes of the blocks determined by the time to fixation and amount of recombination). After this occurs, selection and drift can still occur within ancestry backgrounds, and really the two processes can occur at the same time even. At present, little distinction is made between sorting of ancestry blocks versus evolution with ancestry backgrounds, even after genome stabilization (this is discussed a bit here and there, but it is not as central as it should be). Nor is it clear from this manuscript (perhaps more clear from past work) how much progress has been made towards genome stabilization. I think these issues are critical for interpreting patterns of genetic differentiation in Italian sparrows. For example, tests of selective sweeps in Italian sparrows could pick up signals of past sweeps that occurred in one of the parents if they simply fixed ancestry blocks from that parent. Likewise, tests of selection based on haplotype blocks would presumably be very dependent on whether or not Italian sparrows still have segregating ancestry blocks from the parents in a given region of the genome.

I think this distinction between ancestry sorting during stabilization and selection and drift operating after stabilization needs to be made more clear, including in the conceptual set up (e.g., Figure 1). I think this should then translate into analyses focused on explaining ancestry (e.g., Fst based on ancestry), genetic differentiation within ancestry, and genetic differentiation overall. I realize that hybrid index (which really should be local ancestry I think) is used in the last set of analyses, but I don't think this is sufficient.

**RESPONSE:** We agree that we should be more clear on what is expected from sorting of ancestry blocks, and what is known about the level of genome stabilization in the Italian sparrow. To address this important point rose by Reviewer 1 we have therefore:

1) Added a discussion on genome stabilization in hybrid taxa and its potential effect on genomic differentiation, early in the introduction (Lines 122-128; 164-169; 187-190 and 215-250). We also included an analysis on fixation rate of differentially fixed parental alleles across islands as a proxy for genomic stabilization (please see Lines 659 - 673; Lines 857 - 868; Figure S9 in manuscript and the response to the reviewer’s point 2 below).

We have as well provided information of what is known on the level of genome stabilization in the Italian sparrow, mentioning for instance the fixation of house mitonuclear and DNA-repair loci (Lines 108-111) and the observation of low parental differentiation on highly differentiated loci in Italian sparrows from mainland Italy (Lines 983-986)

2) Added predictions specific to sorting of ancestry blocks. Specifically, we would expect the genome stabilization process to involve sorting of incompatible combinations. This selection to create a compatible, “stabilized” genome would have a disproportional effect on sites that are fixed or close to fixation between the parent species. However, for regions of the genome where the alleles segregate in both parental species, we do not expect that genome stabilization processes are as important, as these alleles are unlikely to cause incompatibilities. Hence, estimating parental *F*_ST_ and identifying sites that are strongly differentiated between the parent species and sites that have intermediate frequencies in the parent species would be informative of whether sites expected to be affected by genome stabilization processes differ to those that are not (e.g. in the slope of Fig. 1A). We have developed this reasoning in the conceptual Figure 1 and its caption as well as in the text in the revised version (Lines 215-250; 931-937).
3) We have also discussed our findings in the context of genomic stabilization and how it could affect the patterns found (e.g. Lines 805-818; 824-855; 931-941).
4) To address the concern that tests of selection based on haplotype blocks would be affected by whether Italian sparrows still have segregating ancestry blocks from the parents in a given region of the genome, we refer to our estimates of fixation rates. In summary, we find that only an average of 5.8% of the fixed differences between parent species are fixed within the island populations (span 17% to 0.3%), consistent with both parental variants segregating for the absolute majority of the genome. We hence do not think that fixation of specific parental haplotypes should have any major impact on haplotype based selection tests.
5) We are grateful for the suggestion to examine ancestry, but it is not immediately clear to us what “F_ST_ based on ancestry” or “genetic differentiation within ancestry” imply in terms of analyses. Would “genetic differentiation within ancestry” correspond to genetic differentiation within islands in regions of a single ancestry based on the LLAP ancestry estimates (Fig. S9A)? If this is the case, we showed that there is indeed genomic differentiation within islands in regions where LLAP ancestry estimates from one population per island suggest either Spanish or house sparrow ancestry, as there was no correlation between LLAP and within-island differentiation (Fig. S9A). There is also no significant correlation between differentiation to either of the parent species and within-island differentiation? If we have still not answered the reviewer’s concern we will be very grateful for a further clarification of exactly which analyses the reviewer is envisioning to address this concern, and we will happily include these it in a new revision of the manuscript.

Finally, to clarify our use of ancestry estimates, we have used whole genome data from Runemark et al (2018) *Nature Ecol Evol* for Crete, Corsica and Sicily, and parental species from Elgvin et al (2017) *Sci Adv* and Ravinet et al (2018) *Proc. Biol. Sci*. using LOTER to generate ancestry estimates, and later calculate a per loci local ancestry proportion (LLAP), previously referred to as Hybrid Index (HI) in the manuscript. While we certainly want to strive to use the best estimates of ancestry, it is not clear to us what method is suggested, but with more clear instructions of software and approach we would be happy to include this in a future revision.

2. Related to point 1, it is not really clear from this paper at least how much progress has been made towards genome stabilization and how independent the islands and populations within islands are in terms of progress towards genome stabilization. There are a few mentions that three independent origins are hypothesized, but not much cited or shown to support this. Knowing how much stabilization has progressed and how independently across islands is important for interpreting patterns of genetic differentiation.

**RESPONSE:** Results supporting the hypothesis of independent hybridization events are presented in Runemark et al (2018) *Nature Ecol Evol*. Following methods from Meier et al., (2017) *Nat. Commun*) the correlation of the *f*_d_ statistic (as a measure of ancestry) for the same windows across the genome was used to test whether Italian sparrow island populations are independent replicates. The rationale here is that common hybridization event would result in a strong correlation in ancestry across islands, whereas low correlations suggest independent origins. Results show the correlations of *f*_d_ ranged between 0 and 0.37 among Italian populations pairs (see Runemark et al (2018) Supplementary Fig 5A presented below (Fig.R2)). We have now explicitly included this supporting information and in the introduction (Lines 254-258) and along the manuscript (Lines 1115-1121).

**Figure R2.** Supplementary Figure S5 from Runemark et al., (2018) Nature Ecology and evolution. Original legend states as follow: *“****Analysis of independent origin based on ancestry blocks. A.*** *Correlation of ancestry blocks among Italian sparrow populations is significantly lower than that among lake Victoria cichlid species representing a much older hybridization event. The boxplot illustrates correlation of f_d_ in 10kb windows between Italian sparrow populations with correlation of blocks inherited from the house sparrow colored in blue and from Spanish sparrow in red. This suggests that the Italian sparrow populations originated from several different hybridization events.* ***B.*** *The size distribution of putative ancestry blocks shows smaller ancestry blocks for Spanish sparrow (red) than house sparrow (blue). Blocks that can not be clearly allocated to either parent species are illustrated in grey. The size distribution of the ancestry blocks is consistent with more recent hybridization events for the Italian sparrow than that of the lake Victoria cichlids”.*

To address whether the island populations differ in degrees of genome stabilization, we have now estimated the rate of fixation of differentially fixed parental alleles (Fig. R3 and Fig. S9 in the manuscript). While hybrid genome stabilization might not necessarily imply fixation, we think that differences in the degree of stabilization would be reflected in the degree of fixation of one of the parental alleles to reduce incompatibility.

We found that Crete shows the highest degree of fixation with a rate of 0.17, while Sicily has a fixation rate of 0.003 and Corsica with a negligible number of fixed loci parentally diverging. When discriminating between each parental ancestry we find, as expected, the ratio of fixed loci in the islands is higher for the ancestry of the major parent. This pattern holds for two out of the three islands. While Crete and Sicily present a higher ratio of fixed loci from the house and Spanish sparrow, respectively. Corsica does not show large values of fixed loci from either of the parents. Among the islands Crete shows the highest rate of fixation (0.168 for house sparrow ancestry). This information is now added in the manuscript (Lines 659 - 673; Lines 857 - 868; Figure S9)

**Figure R3.** Rate of fixation by ancestry in the Italian sparrow. Only sites differentially fixed between parents across the genome were evaluated. Continuity of the y-axis is broken (dashed line) to minimize the size of the figure in order to include the extreme values of the distribution.

3. I am a bit worried that too few SNPs were sequenced for the window-based analyses used here to be appropriate. As best I can tell, 2224 SNPs were used in the analyses (line 494), but maybe that was only true for the Admixture and PCA analyses. I didn't see any other number of SNPs given elsewhere. Assuming 2224 SNPs and assuming a modest genome size for sparrows (e.g., a few hundred Mbs; didn't see the value), this comes out to about 1 or 2 SNPs per 100 kb window. Thus, the windows would mostly be just individual SNPs. And it isn't clear how many of these SNPs are really informative about ancestry. To be clear, I think the number of SNPs is totally sufficient to describe genome-average ancestry as captured by PCA and admixture proportions, and maybe even average ancestry for chromosomes. But I am less convinced they provided detailed information about local ancestry along chromosomes. It appears that additional whole genome data are available (lines 595-596), but were mostly not used here.

**RESPONSE:** While most analyses including all three species are based on the 2224 SNPs from the RAD-data set and analyses where only the Italian sparrow was evaluated were run with a VCF of 2804 SNP; ancestry estimates along chromosomes are calculated using whole genome resequencing (WGS) data of the three species retrieved from Runemark et al. 2018 *Nature Ecol Evol*, Elgvin et al (2017) *Sci Adv* and Ravinet et al (2018) *Proc. Biol. Sci.* Hence, the ancestry estimates are robust and derived from WGS. The genome size of the Italian sparrow is approximately 1GB and the mean number of RAD-based SNPs per window used for FST analyses is of 1,5 (SD: ±0.89), we have now included the mean SNPs per window in Lines 1208. As we do not have whole genome data for all three populations per island (only for one per island), we cannot perform these population genomic analyses based on whole genome data only.

Other detailed comments with line numbers:

L75. Also see <https://doi.org/10.1038/s41467-020-15641-x>, which instead compares ancient and contemporary natural hybrids (and address the role of recombination rate in genome stabilization/ancestry).

**RESPONSE:** We thank the reviewer for pointing out this very relevant reference. We have now included it in the manuscript (Lines 150 and 894).

L86-90. These sentences would be more clear if put in the context of ancestry sorting versus evolution within ancestry types as noted above.

**RESPONSE:** This paragraph has now being modified accordingly (see Lines 162 - 169).

L127. Here, "potentially originating from independent hybridization events" denotes a fair bit of ambiguity about how likely or unlikely this is. But for most of the paper, it appears that independent origins are assumed.

**RESPONSE:** The hypothesis of independent hybridization events is supported by the results presented by Runemark et al., (2018) *Nature Ecol Evol*. Please see our response to the major comment on independence (point 2) above. We have mentioned these results in the manuscript and cited the corresponding study

L139-141. This assumes similar environmental gradients within and among islands, right? If so, maybe make this more explicit.

**RESPONSE:** We modified the hypothesis to make it clearer. Here we are not specifically addressing similar environmental gradients among and within islands, rather we hypothesize that some of the genomic differentiation among islands could be explained by differences in selective pressures among islands, driven by e.g. differences in environmental conditions among the islands.

L152-154. Are these just averages of window-based Fst estimates, or proper, multilocus estimates of overall Fst (i.e., average of ratios or ratio of averages)?

**RESPONSE:** Here we are reporting the average of the weighted windows *F*_ST_ estimates for pairwise comparisons generated by vcftools. We have now modified the sentence to make it clearer (Line 329).

Fig 3A. The legend here needs more information. I suspect the violin plots show the distribution of Fst for windows, but make this clear, including defiing the dashed segments of the vertical lines. And then note that point and bars (presumably) are the mean and 95%CI on the mean.

**RESPONSE:** We thank the reviewer for bringing our attention to this legend. It is indeed lacking information. We have modified it now making it more informative.

L156-157. One can get this from the figure, but how much higher? An effect size estimate would be more interesting than just giving results from a null hypothesis significance test.

**RESPONSE:** We have now performed the effect size estimate for this comparison, finding a medium Cohen's d of 0.523, with 95% confident intervals of 0.49 - 0.56. These results are now included in the manuscript (Lines 339-341). To clarify our previously reported statistic, we initially ran a Monte-Carlo test with 10.000 permutations and later report a Bonferroni corrected p-value.

Table 1. Maybe I am missing something, but the last row of the table suggests that 99.8% of variation within individuals is not significantly different from 0. Is that the correct interpretation?

**RESPONSE:** That is correct. In this case % of covariance within individuals refers to heterozygosity. Here we find that variation is not significant as per the Monte Carlo randomization by permutation. Permutation results are also presented in supplementary figure S1.

L196. I don't think it is okay to equate divergent selection with the results of a specific test for selection, in this case xp-EHH. Speaking of correlations between Fst and xp-EHH is fine, just don't call xp-EHH divergent selection. And as noted above, I am concerned that this haplotype-focused tests might be affected in odd ways by (partial) genome stabilization, and also by the low density of SNPs (assuming just 2224 across the genome). Lastly, the very low r2 values for these tests should be highlighted (i.e., yes these metrics are correlated, but Fst explains almost none of the variation in xp-EHH).

**RESPONSE:** We agree with the reviewer that Cross Population Extended Haplotype Homozygosity (xp-EHH) is not per se divergent selection. We have now rephrased the sentence and also acknowledge the low R^2^ of the model (see Lines 439-442).

By comparing haplotype lengths this statistic allows us to identify selective sweeps present in only one of the populations included in the pairwise xp-EHH comparison. Thus we can detect genomic regions under positive selection diverging between islands. Even though, as the reviewer pointed, RAD data doesn’t have the same power as WGS data, there are several examples where selective sweeps statistics from RAD data is still very informative, e.g. Roesti et al., (2015) Nature Communications (<https://www.nature.com/articles/ncomms9767?message-global=remove>) and Marques et al., (2016) PLOS Genetics. (<https://journals.plos.org/plosgenetics/article?id=10.1371/journal.pgen.1005887>)

However, to address the reviewer concern regarding the low power of RAD data when estimating xp-EHH we explored a signal of extended haplotype homozygosity in chromosome 2 (111:113Mb) previously identified in the house sparrow using whole genome sequencing data by Ravinet et al., (2018) *Proc B* (see Fig R4). Using the RAD data we can also detect higher values of xp-EHH on this site when comparing Italian sparrows between islands and between parental species (Fig R5A), as well as in some comparisons of the Italian sparrow and its parents (Fig R5B). If the editor recommends so, we could of course include this results as supplemental material.

.

**Figure R4.** Sections of Figure 3 from Ravinet et al., 2018 Proc B, showing peaks of divergent haplotype homozygosity (xp-EHH) occurring in chromosome 2.

**Figure R5.** xp-EHH across chromosome 2 showing a peak around (111:113Mb) previously identified in the house sparrow by Ravinet et al., 2018 Proc B. **A**. comparing Italian sparrows between islands and between parental species B. as well as comparisons of the Italian sparrow and its parental species.

We also acknowledged that R^2^ of the FST vs xp-EHH comparisons are small and emphasized this issue in the text (see lines 445-447) and include it in the Fig.5A

L236-237. Again, how much higher? How big is the effect? Without an effect size, P-values don't really tell you much.

**RESPONSE:** We thank the reviewer for spotting that we have only reported the *X*^2^ values in the supplementary table and not in the text. We now have included the statistic in the text and run size effect analysis.

L253. How well explained? What is the r2?

**RESPONSE:** We have now included the multiple R2 of the model, which include all the predictors, where differentiation against the Spanish sparrow (FST) and recombination rate significantly explained the genomic differentiation within Corsica and within Sicily, respectively. Estimates for each predictor within the model are also reported in the text (see Lines 596-619) and Table S8.

L292. First, this should I think be local ancestry not hybrid index. Hybrid index is normally used to refer to average ancestry for an individual, not average at a locus across individuals. Unless the former is what this metric is. Second, one might expect higher differentiation not based on local ancestry per se but z (1-z) (where z is local ancestry), that is, intermediate versus extremes not one parent type versus the other.

**RESPONSE:** We have now changed the term “Hybrid index” to “per locus local ancestry proportion (LLAP)”. This metric looks into the proportion of either house or Spanish ancestry in a specific block across all individuals of a single island. Thus, ancestry estimates (retrieved by LOTER) will be determined per locus per individual, LLAP is calculated per locus as the proportion of the frequency of house ancestry / frequency of Spanish ancestry across all individuals of a specific Island. The main reason why we are interesting in comparing proportion of house vs Spanish ancestry was to be able to differentiate the effect of ancestry from the minor- vs. the major-parent. Analyzing the extremes vs. intermediate ancestries, as the reviewer suggest, would inform us whether blocks that present ancestry from both parents, segregating in the hybrid (intermediate) or those close to fixation for either of the parents (extreme) are overrepresented in the highly differentiated loci within an specific island, contributing to genomic differentiation across populations, which is an interesting question, but it wouldn’t enable us to know if there is an important effect of those loci specifically inherited from the minor- or the major-parent.

L310. Fine, but isn't this just the basic IBD signal one expects to find in almost any system?

**RESPONSE:** We agree the reviewer with this observation, as populations among islands are isolated you would expect a higher differentiation among islands than between populations within an island. We have modified the paragraph accordingly. However it is important to clarify that there is not a clear tendency of IBD among the islands. The most distant islands (Corsica and Crete) do not show the highest *F*_ST_ in comparison with the other pairwise comparisons (Cosica vs Sicily AND Sicily vs Crete). *F*_ST_ between Corsica-Crete has a similar value as the Crete-Sicily *F*_ST_.

L315-316. What might explain the observed pattern? This is another case where ancestry thinking and progress towards genome stabilization could be useful.

**RESPONSE:** We thank the reviewer for bringing our attention to this and have now modified the paragraph addressing genome stabilization on the context of divergent selective sweeps among islands and local selection patterns within islands (see Lines 805-818)

L319. Results from one test are not "patterns of selection".

**RESPONSE:** We agree and have now changed this sentence accordingly.

L448-450. Citation to support this? And maybe summarize past evidence along with citation.

**RESPONSE:** We have now added the citation and a few sentences specifying the results that support the scenario of independent hybridization events (see Lines 1115-1121).

L474. I think phred 20 is 99% confidence, not 99.9%

**RESPONSE:** We thank the reviewer for spotting this mistake and have fixed this now.

L479. Is `max-missing' allowing for 80% of individuals to be missing data (no genotype)? If so that is really high and should be acknowledged and justified. And define what each parameter is.

**RESPONSE:** We apologize for this misunderstanding. Vcftools is very confusing in this way but it is actually the opposite – 0 means individuals with all missing data are allowed, 1 means no missing data is permitted. Thus 0.8 means we tolerated only 20% missing data for an individual/site, we clarified in the sentence (Line 1161). Filtering using a 20% missing data threshold was done only on the raw VCF files that contain ~1.9 million SNPs, before filtering by genotype quality, depth of coverage, per SNP missing-ness and minor allele frequency. Thus, this per individual missing-ness threshold is only intended for identifying and excluding those individuals with very little information at an early stage. These removed individuals are not included in the manuscript. The reported individuals in the Table S1 and in the manuscript are those that passed this early stage filter. However, to address this concern we calculated per individual missingness in the final VCF files. For Spanish-only and house-only VCF files there are no individuals with missingness greater than 3%. For the VCF file containing only Italian sparrow 2.2% of individuals (5 out of 223) have missing data greater 50% and an overall mean missingness of 13%. For the VCF that contains all individuals of the three species (house, Spanish and Italian sparrows), this accounts for 11 out of 315 individuals, and an overall mean missingness proportion of 13%. And for the VCF with all Italian sparrows and the reference parental populations this accounts for only 2% (5 out of 244) of individuals. We now included this information in the Methods section (Lines 1165-1166).

For the VCF with all three species we provide a table specifying those individuals of missing data proportions >50% and a histogram showing the distribution of missingness across all individuals (see Table R2. and Fig. R6 below). A new PCA analysis was also performed removing those individuals with high % of missing data described above and no major changes are seen between this and our original PCA retaining these individuals (see Fig. R7).

**Table R2.** Proportion (F_MISS) of missing per individual larger than 50% in the VCF file containing **A.** all three sparrow species.	**Figure R6.** Histogram of the proportion of missing data per individual for the VCF containing all 3 focal species.

**Figure R7.** Assessing the effect of a 3% missing-ness. PCA performed after removing individuals containing missing data > 50% in the 3 species dataset.

L480-481. This could be a bit more clear. Are SNPs with r2 > 0.1 within 25 bp or 100 kb removed?

**RESPONSE:** We have now clarified this filtering step in the manuscript. We calculated linkage disequilibrium in sliding windows of 100kb in size and with a step of 25bp, then pruned sites with a r2 score > 0.1.

L487. Capitalize `component' for consistency.

**RESPONSE:** It’s now fixed.

L494. The number of SNPs should be in the Results too. I don't think you want to bury the fact (I think anyway) that the whole paper is based on 2224 SNPs.

**RESPONSE:** We have now included the number of SNPs use for the PCA and Admixture analysis in the figure legend as well as in most of the other figure legends. We also include it in the description of these and other statistics (e.g see L325, 329, 342). Other analyses are based on VCF files containing between 2856 and 2804 SNPs, (i.e. only Italian sparrows VCF, Italians + reference parental individuals VCF and only parent VCF). Initially we also provided a summary of the VCF files used in each analysis in the supplementary table S2.

L519-526. I think the description here is very clear, more so than in the main text of the Introduction and Results.

**RESPONSE:** We have now modified our explanation of the tests in the results and the conceptual background of it in the introduction (see L184-196 and 361-388)

L530. How many SNPs on average per window?

**RESPONSE:** On average the number of SNPs per window for the windowed-based FST estimates is of 1,5 (SD: ±0.89).

**Reviewer #2:**

In their manuscript entitled ‘Predictors of genomic differentiation within a hybrid taxon’ Cuevas et al. sequence a series of populations on three islands of a homoploid hybrid species, the Italian Sparrow, and use various population genomics methods to compare differentiation within and between islands, as well as between hybrids and the two progenitor species to better understand what factors influence the resolution of hybrid genomes. Specifically, they aim to understand whether differentiation (measured by Fst) within hybrids is a factor of recombination, selection, or the major/minor ancestry of that hybrid population. I think this work is both timely and very important for our field, and I agree that the authors have a powerful system to address these questions.

Overall, I quite enjoyed the manuscript and the results are both intriguing and novel, in my opinion. However, there are many parts of the manuscript that I found quite confusing and I’m not entirely sure that I agree with some of the predictions that the authors lay out as uniquely supporting certain evolutionary scenarios. In this light, I have a few suggestions for clarification throughout the manuscript, as well as concerns that the authors may wish to address in revision.

1. This may be picky, but I feel the authors use the terms ‘constraint’ and ‘contingency’ somewhat differently than how I think the majority of evolutionary biologists use these terms. In my mind, constraint is a product of substantial purifying selection constraining a sequence from evolving (and therefore it is conserved across time). The authors seem to be using this term more so describe a scenario in which hybrid genomes resolve in similar ways (e.g. the same parental allele fixes or rises in frequency), potentially due to the fixation of interacting incompatibilities at other loci. To me, there are two very separate processes. Similarly, although to a lesser extent, it seems that the authors use ‘contingency’ to describe scenarios under which regions of the genome in hybrids resolve to look like one parent, and because of epistasis, this influences the probability that loci elsewhere in the genome will also resolve in the same direction (e.g. the fate of an allele at one locus is defined by the fate of alleles at other loci). While I don’t think this is necessarily entirely out of the realm of how researchers of adaptive landscapes and protein evolution might think of evolutionary pathways from one multi-locus genotype to another (for example, how Lewontin might have envisioned this path), this terminology usually refers to the evolutionary order of mutations, not the resolution of pre-existing variation. Again, I think this is a picky point, but I would encourage the authors to be very explicit about how they are using these terms in their introduction. It would then also help to clarify comments like “What is the relative importance of selection vs. constraints in this process?” (lines 22-23), as really both of the processes the authors are referring to are caused by selection (one being potentially genotypic novelty that facilitates novel adaptations, the other being the removal of incompatible combinations).

**RESPONSE:** We are very happy that Reviewer 2 enjoyed the MS and found the results intriguing and novel. In the revised version, we have done our best to clarify the question marks raised. We have also gone over the terminology to ensure that concepts are not confounded (e.g. Lines 128-131). The part on the relative importance of selection and constraints has been rewritten in response to this and to comments about disentangling contributions of hybrid genome stabilization and other processes (e.g 164-169; 187-190 and 215-250 among other lines across the manuscript.

2. A major goal of this work is to determine if hybrid genomes are more strongly influenced by selection against incompatibilities versus natural selection via adaptations. I think this is a question of high significance for our field, however, I’m not convinced that the predictions outlined in Fig. 1 uniquely support some of the scenarios that the authors suggest they might, and I think there are some underlying assumptions for some of these predictions that are not clearly laid out by the authors. I address these concerns in the context of each prediction/panel.

**RESPONSE:** We thank the reviewer for this observation and we agree that the description of the hypotheses in Figure 1 should be clarified not to be misinterpreted as if they were uniquely supporting the different scenarios; we are proposing them as potential indicators of the evolutionary processes that could affect patterns of differentiation in a hybrid lineage. We have now made major changes in the caption of the figure and the corresponding description in the introduction.

**- Fig. 1A:** One potential issue and one very nit-picky comment:

* Larger issue: Because the authors are using differentiation, not minor parent ancestry, I’m not entirely convinced that the reduction in differentiation in hybrid populations with increased recombination is uniquely showing the removal of incompatibilities. In my mind, the reason why this scenario is so plausible in, for example, Schumer et al. 2018 Science, is because regardless of who the minor parent is, minor parent ancestry is much reduced in low recombination regions, and this uniquely supports the removal of incompatibilities (versus say, removal of slightly deleterious variation). In this present analysis, while the authors also have hybrid populations that differ in what parental species is the major/minor contributor, they instead use Fst between hybrid populations, with the expectation that low recombination regions should show less differentiation than differentiation between the parents. I think there are two issues with this interpretation: 1) This type of analysis assumes that incompatibilities are resolved in the same way between hybrid populations. This is probably not unreasonable, because the hybrid populations being compared here are from the same islands and have roughly the same major/minor parental contributions (within islands). However, this is never explicitly stated as an assumption (although it is hinted at throughout the introduction). But, even still, I am not sure that this relationship uniquely supports removal of incompatibilities- which leads to my second concern: 2) this pattern could also be caused by scenarios in which those hybrid populations are adapting to similar habitats, and/or those hybrid populations are purging the same mildly deleterious alleles inherited from one parent. In all cases the same parental ancestry is removed and hybrids will show less differentiation. I might suggest that the authors further test this prediction by looking at minor parental ancestry across recombination bins for all 9 of their populations (as in Schumer et al. 2018. Science. Another recent example of this type of analyses is performed in Calfee et al. 2021. Selective sorting of ancestral introgression in maize and teosinte along an elevational cline. BioRxiv).

**RESPONSE:** We thank Reviewer 2 for bringing up these points. We agree that our underlying assumptions for figure 1 should be clarified, and have now done so in a revised version of the MS in the introduction (164-169; 187-190) and in the result section where we test for this hypothesis (Lines 361-388).

First, to clarify the point regarding resolution of incompatibilities *within-* and *among-*islands, as the reviewer mentioned, the estimation of genomic differentiation in this case is *within*-islands, thus we do expect that genome stabilization within islands results in similar resolution of incompatibilities given the similarity in ancestry proportions within islands. Hence, the comparisons in Fig. 1A where we present *F*_ST_ among populations within islands would not reflect if the major parent ancestor is different for the three islands. Instead, purging of minor parent alleles would result in reduced variation within the island regardless of which is the majority ancestor. We have now clarified that *the prediction is valid for comparisons within islands* given the potential similar resolution of incompatibilities (Lines 215-250).

Second, we would not expect that neither similarity in selection pressures nor deleterious variation in one parent species would have a disproportionate effect in low recombination areas. To result in a pattern where differentiation is lower in low recombination rate regions, it would require stronger concordance in ecological selection for genetic variation found in low recombination areas compared to the genome wide average, or faster accumulation of deleterious genetic variation in low recombination regions, which there is no empirical evidence to support to our knowledge.

To test the possibility that parallel selection can occur and have similarly affect on patterns of differentiation as constraints would, we also performed correlations of *F*_ST_ across all possible comparison *within-* and *among*-islands, please see the response to the reviewer’s question regarding **Fig. 1B** below. Additionally, we would also like to bring the reviewer’s attention to the *within-*island FST comparisons among islands we previously performed, reported in Figure S4, where we found that overall levels of population differentiation within islands vary between islands.

Hence, we are confident that the pattern described in Fig. 1A indeed would be indicative of selection against minor parent ancestry in low recombination regions. We do, however, agree that our sampling design allow us to test if minor-parent ancestry blocks is reduced in regions of low recombination, a hypothesis previously presented in other study systems by Schumer et al. (2018) *Science* and Calfee et al. (2021), among others. We have performed these analyses, which have inconclusive results, not adding further evidence in any direction, and present them here for review. However, if the editor recommends so, we could of course include them as supplemental material.

Please find the **ancestry vs. recombination** analyses performed to address whether minor ancestry parent is reduced from low recombination regions below:

Ancestry estimates were calculated by LOTER, using WGS data from Ravinet et al (2018), Elgvin et al (2017) and Runemark et al (2018) of 2 reference parental populations and the islands populations. As mentioned in the manuscript, the estimates of ancestry by haplotype generated by LOTER are more reliable if calculated using whole genome re-sequenced data (WGS) than RAD-seq, therefore we used the WGS data available in previous studies. As we do not have whole genome data for all three populations per island (only for one per island), we cannot perform these ancestry estimates across all populations within islands. However, using a single population per island a per locus local ancestry proportion - LLAP (previously called Hybrid index) was generated, where values of 0 correspond to loci where only Spanish ancestry is present across all individuals in the island evaluated and 1 corresponds to pure house sparrow ancestry. We estimated the LLAP separately for each island. LLAP values corresponding to the minor-ancestor per each island were correlated to the recombination rate.

Pearson correlations and linear models for all values of recombination rate as well as binning recombination rate in quartiles were run (See Table R3, Fig. R8). While we find that Corsica present a significant positive relationship between minor-ancestry and recombination rate (more minor-ancestry blocks found in higher recombination regions), the other two islands do not present conclusive results as their relationship appear negative. Moreover, the variation explained on minor-ancestry frequency by recombination rate is considerably low (adjusted R^2^ < 0.008, Table R3).

**Table R3. Linear models of recombination rate and minor-parent ancestry across islands.** The models are performed using values of Log10 of recombination rate as a predictor of local ancestry (LLAP) and dividing these in quartile bins to group the recombination rate values and facilitate the comparison with results presented in other studies.

| **Linear model**  Using Log10 of recombination rate | **Estimate** | **Std. Error** | **Adjusted R2** | **p-value** | **Island** |
| --- | --- | --- | --- | --- | --- |
|  | 0.081955 | 0.011840 | 0.0001975 | 4.47e-12 | Corsica |
|  | -0.028072 | 0.009421 | 2.673e-05 | 0.00288 | Crete |
|  | -0.272958 | 0.004473 | 0.008688 | <2e-16 | Sicily |
| **Linear model**  using quartile bins of Log10 of recombination rate | **Estimate** | **Std. Error** | **Adjusted R2** | **p-value** |  |
|  | 0.0012136 | 0.0001036 | 0.0005732 | < 2.2e-16 | Corsica |
|  | -0.0005305 | 0.0001072 | 7.969e-05 | 7.47e-07 | Crete |
|  | -0.0088202 | 0.000148 | 0.008203 | < 2.2e-16 | Sicily |

**Figure R8.** Proportion of minor-ancestry (using LLAP, where values of 1 = 100% house ancestry and 0=100% Spanish ancestry) vs recombination rate presented in quartiles using whole genome resequencing data retrieved from Ravinet et al (2018), Elgvin et al (2017) and Runemark et al (2018). Mean and confident intervals of LLAP are shown per recombination rate quantile. Minor ancestor re as follow: **Corsica:** minor-ancestry of the Spanish sparrow (LAAP= 0 to 0.5), n=237.523 SNPs. **Crete:** minor-ancestry Spanish sparrow (LAAP= 0 to 0.5), n=294.749SNPs and **Sicily:** minor-ancestry the house sparrow (LAAP= 0.5 to 1), n=424.739SNPs.

* Smaller point: Should the green and purple lines actually cross in this panel? I agree that the slope of the green (hybrid-hybrid) line should be flatter than the purple (parent-parent) line, but under what scenarios do the authors envision that hybrid-hybrid comparisons should show higher differentiation than parent-parent- comparisons (e.g. that the purple and green lines cross)? I suppose this could be the case in scenarios in which some hybrid populations are adapting to a completely novel environment and are therefore experiencing very strong divergent selection (more so than between parents), but otherwise I would expect that the differentiation among hybrid populations is probably capped at the differentiation among parental species.

**RESPONSE:** We thank the reviewer for this observation. We agree the lines should not cross as in the extreme case of purging all minor-parent ancestry in low recombination regions would in fact equal the variation to within one of the parental species. We have now corrected the figure.

**- Fig. 1B:** it is not clear to me why parallel genomic signatures would be expected under scenarios of adaptation both within and between islands, unless the adaptation is parallel within and between islands. Or, are the authors invoking widespread background selection? Because of the sampling, the authors might be able to differentiate these scenarios: If you have a scenario in which island A has populations a’ and a, and island B has populations b’ and b, you might expect parallel genomic differentiation if populations a’ and b’ are both adapting to the same environment (e.g. there is strong parallelism in selection) and this parallelism affects the vast majority of the genome. In which case, you’d expect parallelism between the a vs a’ and b vs b’ contrast, and also between a vs b’ and a’ vs b contrast, but not the others. Under background selection, one might expect all contrasts to have correlated signatures. Have the authors done these types of analyses to contrast evidence for background selection or parallel ecological adaptation? Just one final note on this type of analyses: in my experience, these whole-scale genome correlations in Fst are very common, especially between recently diverged populations, and they are usually due to shared ancestral variation. In my mind, there is still debate as to whether these correlated landscapes are due to shared adaptive evolution or background selection, with evidence on both sides. For example:

Burri et al. 2015. Linked selection and recombination rate variation drive the evolution of the genomic landscape of differentiation across the speciation continuum of Ficedula flycatchers. Genome Research (which the authors already cite)

Stankowski et al. 2019. Widespread selection and gene flow shape the genomic landscape during a radiation of monkeyflowers. PLoS Biology.

**RESPONSE:** We agree that it is important to differentiate background selection and adaptive parallel selection, and thank Reviewer 2 for pointing this out. Due to multiple population pairs being present, we are in a unique position to differentiate between background selection, expected to be present in all comparisons, and parallel selection, present in some but not all comparisons. We have now performed additional comparisons to differentiate between background selection and parallel selection. We find variation in the strength of correlation of differentiation among subpopulations comparisons. This pattern is consistent with some degree of parallelism in selection rather than background selection, as we would expect differentiation to be correlated across all comparisons in case background selection strongly limits which areas of the genome are free to vary. However, neither of these forces strongly affected the distribution of differentiation, as none of the comparisons were significant when correcting for multiple testing (Fig. R9, Fig. R10, Table R4). These results are now incorporated in the manuscript (Lines 523-579; 902-925; 1289-1295; Figure S6, Figure S7, Table S13 in the manuscript)

**Figure R9. Parallelism of *within*-island pairwise FST**. Pairwise FST between populations within island “A” are correlated to pairwise FST estimates of populations within island “B”. Pairwise correlations of within-island FST in island A vs. within-island FST in island B are highlighted in green and those significantly correlated are depicted in red. Abbreviations of the comparisons are as follow:

CORSICA populations: Muratello (Mur), Pianiccia (Pi), Tiuccia (Pi). CRETE populations: Istro (Is), Mithimna (Mi), Perama (Pe). SICILY populations: Cos (Co), Enna (En), Naxos (Na). Thus pair-wise *F*_ST_ between Muratello vs. Pianiccia is abbreviated as “*Cor_Mur.Ti*”. Similarly, pair-wise *F*_ST_ between Enna vs. Naxos is abbreviated as “*Sic_En.Na*”

As the reviewer suggested we evaluated correlations between genomic differentiation (*F*_ST_) of the a vs a’ populations of island A and the *F*_ST_ of b vs b’ from island B (Fig. R9). Of those significant contrasts found when comparing *within*-island *F*_ST_ we checked for parallelism in differentiation across islands by comparing genomic differentiation (*F*_ST_) between the a vs b’ and a’ vs b contrast, as it could be expected if parallel differentiation is occurring across subpopulations of the different islands.

We find some evidence of parallel genomic differentiation as it could occur if a subpopulation within one island is under similar adaptive selective pressures as those of a subpopulation on a different island. Some pair-wise comparisons of subpopulations within Sicily and Corsica are correlated as well as two comparisons of subpopulations within Sicily and Crete, but this is not the case for the comparison between Corsican and Cretan populations (Fig. R9).

However, when correcting for multiple testing by resampling and running 100 iterations of the correlations we find that none of the parallel significant within-island correlations hold significance (see Table. R4)

| **comparison** | **t-estimate** | **df** | **correlation** | **p-value** |
| --- | --- | --- | --- | --- |
| Sic_Cos.En vs. Cor_Mu.Ti | 1.681115 | 1498 | 0.04 | 0.13 |
| Cor_Pi.Ti vs. Sic_Cos.En | 1.681115 | 1498 | 0.04 | 0.13 |
| Cor_Pi.Ti vs. Sic_Cos.Na | 1.529509 | 1498 | 0.04 | 0.17 |
| Cor_Pi.Ti vs. Sic_En.Na | 2.395912 | 1498 | 0.06 | 0.05 |
| Cre_Mi.Pe vs. Sic_Cos.En | 2.110884 | 1498 | 0.05 | 0.08 |
| Cre_Is.Pe vs. Sic_En.Na | 1.820645 | 1498 | 0.05 | 0.11 |

**Table R4.** Mean estimates after 100 iterations of correlations. *P* values, correlation values and t-estimates are corrected for multiple testing by resampling and taking mean estimates of 100 correlations.

Nonetheless, after correcting for multiple testing we still wanted to evaluate correlations of individual subpopulations across islands. Similarly, these individual subpopulations also show a similarities of *F*_ST_ distribution across islands, meaning significant correlation (prior correction) of a-b *F*_ST_ vs. a’-b’ *F*_ST_ as well as correlation of the a-b’ *F*_ST_ vs. a’-b *F*_ST_ comparison (Fig. R10).

**Figure R10. Parallelism of *between*-island pairwise *F*_ST_ across all subpopulations.** Correlations of pairwise-*F*_ST_ between subpopulation a (from island “A”) and b (from island “B”) and its contrast pairwise-*F*_ST_ between subpopulation a’ (from island “A”) and b’ (from island “B”). **A.** Correlations between Corsican vs. Sicilian subpopulations. **B.** Corsican vs. Cretan subpopulations and **C.** Sicilian vs. Cretan subpopulations. Populations’ name of each island are presented in Figure X1. Abbreviations of the comparisons are as follow: As an example, pair-wise *F*_ST_ between Muratello (from Corsica) vs. Enna (from Sicily is abbreviated as “*Cor.Mur_Sic.En*”. Similarly, pair-wise *F*_ST_ between Perama (from Crete) vs. Naxos (from Sicily) is abbreviated as “*Cre.Pe_Sic.Na*”

Lastly, If the authors think that this parallelism might be caused by replicated differences in ecology, could they expand on this hypothesis in the introduction? Is there some sort of replicated environmental gradient on these islands that might drive these patterns?

**RESPONSE:** While parallelism in environments in response to genomic differentiation for loci involved in adaptation to a specific environmental factor is an interesting topic, it is not within the scope of the current study. The study design is, while well adapted to address questions about background selection as explained above, not well suited for this purpose as we lack parallel environmental gradients enabling comparisons of genomic parallelism in response to these.

**- Fig. 1C**: Again, the logic of this a bit confusing to me, and I don’t think these patterns unique support a role for incompatibilities: highly differentiated regions between parental species should include incompatibilities (as the authors suggest), but also many regions potentially involved in adaptation. If different hybrid populations are adapting to the same environment (and this happens to mirror an environment experienced by one of the parents), then Fst between hybrids might be reduced at regions of the genome that are highly differentiated among parents. In addition, and as stated above, there’s also no reason to expect that different hybrid populations will resolve all incompatibilities in the same direction, and therefore no reason to expect that regions harboring incompatibilities will show reduced differentiation in different hybrid populations. As the authors know- and as they lay out in their prediction (V), the likelihood of incompatibilities being resolved in the same direction between populations is strongly related to the major ancestry proportion, but I don’t feel this information is well integrated into this figure, and I think this points gets a bit muddled in the intro and discussion.

**RESPONSE:** We agree with the reviewer that highly differentiated regions between parents could also harbor potential regions for local adaptation as well as incompatibilities. We also agree in that there is not reason to expect similar resolution of incompatibilities in independent populations that differ in ancestry proportions. Our initial hypothesis was intended to tackle *within-*island differentiation where similar resolution of incompatibilities is highly likely. Taking into account the points made by the reviewer we now have modified the figure and clarified the hypothesis leaving room for the different expected pattern for *within-* and *among-*islands differentiation (see Figure 1C and figure legend; Lines 215-250; 968-978).

Moreover, to address the possibility that parallel selection can occur and differentiate it from background selection we have now performed correlations of *F*_ST_ across all possible comparison within- and between-islands (see previous response above).

3. Aspects of the framing of this manuscript seem a bit off to me. For example, framing of adaption in hybrids being facilitated by unique combinations of alleles is not particularly borne out in the authors’ analyses. While there is some evidence of shared outliers in some analyses and in some cases Fst is correlated with other population genomic signals of selection, the authors neither test nor explicitly show that hybrids experience selection for unique combinations of alleles, particularly combinations that involve alleles from both parental species.

**RESPONSE:** We are referring to the potential for the genome to combine in many different ways that *may enable* adaptation, while not specifically testing if adaptive combinations have arisen. We have now rewritten the introduction to reflect this.

4. I think it would be a help to the gentle reader if the authors provided a bit more sign posting and explicit motivation behind their analyses in the results section- especially in sections III, IV, and V. These sections contain a lot of different models that analyze the same data in very subtly different ways, and there are a lot of instances of pairwise correlations between populations at different scale of sampling. Even just a simple sentence or two throughout these sections might greatly help guide the reader to understand why each analysis was performed and what each analyses is telling us (the authors do this in some places already, I just recommend a bit more).

**RESPONSE:** We have strived to consistently include clear motivations in the revised version, and thank Reviewer 2 for this suggestion.

5. The finding that some outliers are shared among comparisons is quite interesting. It might be useful if the authors provided a list of genes within the shared outliers in the supplement, especially if there are any genes of interest. Could the authors also provide an explanation for why outliers may be shared between Sicily and Corsica but not with Crete (whether it be by independent, parallel selection or low level gene flow or some combination). The authors also find that differentiation to the House sparrow genome is negatively correlated with differentiation within islands, suggesting that Spanish sparrow ancestry may be purged in these islands. The authors discuss mito-nuclear co-adaptation, adaptation to human commensalism, and other potential hypotheses of interest in the discussion, but might this pattern also be caused by the Spanish sparrow having a lower effective population size and thus harboring some mildly deleterious alleles, and therefore, these alleles can be recognized and eliminated by selection in a hybrid background?

**RESPONSE:** We have now included a list of genes around 100kb flanking from shared outlier loci (see Supplementary Table S12).

We do find shared outlier loci among all three pairwise comparisons, however, the shared loci between Crete and Sicily do not differ from the neutral expectation (see Lines 480-481). One plausible explanation is the closer similarity on ancestry blocks inherited from the house sparrow on both Crete and Corsica in comparison to that in the Sicilian populations. Moreover, this could also be due to the contribution of *P. domesticus biblicus*, a house sparrow subspecies to the population on Crete (Ravinet et al., in prep) (see Lines 872-879).

It is correct that the nucleotide diversity overall is lower for the Spanish sparrow Elgvin et al 2017 *Sci Rep*, Cuevas et al 2020, *Mol Ecol*), as well as estimates of Ne (Ravinet et al 2018 *Proc Biol Sci*), which could be consistent with a higher incidence of fixation of mildly deleterious alleles. We agree that this should be discussed and have now done so (see Lines 1070-1072).

6. This is more of a minor point, but there are several spots within the manuscript where the authors simply say that two variables are correlated or related, but do not necessarily say that the two variables are negatively related (with the exception of the test statistic being negative). For clarity and to decrease reader confusion, I strongly recommend the authors denote directionality of these relationships when referring to them in text.

**RESPONSE:** This has now been fixed. We thank the reviewer for bringing this to our attention.

Fig. S2/lines 206-209- do the authors think that the lack of correlation between within island Fst and iHS may more strongly point to background selection rather than adaptation and sweeps within islands?

**RESPONSE:** As suggested by the reviewer we evaluated parallel patterns of genomic differentiation, and found no evidence consistent with background selection occurring across all populations within the different islands.

Fig. 5C- are there any outliers shared in all 3 (for both sets of comparisons)? Particularly for the within-island outliers, this might provide evidence for adaptive parallelism. If no, I would denote ‘0’ in the centers of the Venn diagram.

**RESPONSE:** This is a good point- there are no outlier loci shared among the three islands. The figure has now been corrected.

Fig. 4,5,6- can the authors add the correlation coefficient to these scatter plots? It is very hard to tell by looking at them whether the correlations (when they exist) are positive or negative.

**RESPONSE:** Coefficients of significant correlations have now been added to the plots.

Table 2- can the authors list the island term in the top part of this table as well as the Estimated Marginal Means?

**RESPONSE:** This has now been corrected.

Line 24 I think ‘the result’ should be ‘resulting from’

**RESPONSE:** We changed the wording.

Line 25- “we investigated whether different combinations of parental genomes can further evolve”- I think the authors are more asking how and how much have the hybrid lineages evolved, not if.

**RESPONSE:** The sentence has now been modified.

Line 51- incompatibilities can also be ancestral-derived (if the derived allele has experienced more than one mutation). I’d just say incompatible allelic combinations or similar.

**RESPONSE:** We have now rephrased this sentence.

Line 57- these are not a hybrid species, rather just hybrids in a hybrid zone.

**RESPONSE:** This has now been corrected.

Lines 64-66- “These findings suggest that only specific combinations may be viable in a hybrid lineage or only specific portions of the genome are free to vary, potentially resulting in convergent genomic compositions of hybrid lineages.” The wording here is slightly off- under a model of incompatibilities, either parental allelic combination should restore fitness, so this would not necessarily lead to convergence, unless the hybrid populations had a bias in parental ancestry in the same direction.

**RESPONSE:** We thank the reviewer for this suggestion. We have now modified the paragraph (Lines 113-118) to clarify our message on how BDMI could limit variation leading to the fixation of parental allelic combinations in the hybrid, but also acknowledged that there are different solutions to incompatibilities.

line 85-86- “Moreover, linked selection is more efficient in low recombination regions” I wouldn’t necessarily say linked selection is more efficient in these regions per se, but rather that linked selection has a greater impact/influences a larger proportion of the genome.

**RESPONSE:** We agree, and have now modified this sentence to reflect this.

Lines 89-92 in the evidence for selection against introgression in low recombination regions, I would recommend also citing:

Braindvain et al. 2014. Speciation and introgression between Mimulus nasutus and Mimulus guttatus. PLoS Genetics.

As well as a much more recent article:

Nelson et al. 2021. Ancient and recent introgression shape the evolutionary history of pollinator adaptation and speciation in a model monkeyflower radiation (Mimulus section Erythranthe). PLoS Genetics.

**RESPONSE:** We thank the reviewer for bringing our attention to these references. They have now been included.

Fig. 3B- some of the colored writing in the DAPC is hard to read- especially the yellow ‘Enna’. Could the authors use black, but perhaps color the sampling locals in Fig 3C to match the points in the DAPC?

**RESPONSE:** We have now modified the colors of the text within the DAPC.

Line 176- should ‘relation’ be ‘relationship’?

**RESPONSE:** Indeed. This is now fixed.

Line 259-260- “For Corsica and Sicily, there is a significant correlation between within-island differentiation and that against the house and Spanish sparrow, respectively”. However, Fig 6 shows correlation between within Corsica and Corsica-Spanish, and within-Sicily and Sicily-House. Should these be flipped?

**RESPONSE:** We thank the reviewer for spotting this mistake. This has now been fixed.

Lines 311-317: the observation that genomic differentiation within islands is negatively correlated patterns of selective sweeps is not very well explained, except to say this is inconsistent with a model in which standing genetic variation among parents contributes to adaptation in the hybrids (at a genome-wide level). This perhaps seems more consistent with background selection or universal purging of mildly deleterious alleles. Either way, I think the authors could elaborate on this finding.

**RESPONSE:** We have expanded our interpretation of the results by further hypothesize on how genomic stabilization in the hybrid may have an effect on the relationship found between genomic differentiation and signals of selective sweeps (See Lines 807-817). However, we disagree on the possibility that background selection could inflate the haplotype frequency spectrum affecting estimates of positive selection. Harris et al., Genetics (2018) (<https://www.ncbi.nlm.nih.gov/pmc/articles/PMC6283157/>) showed that background selection, which would reduce linked neutral diversity, does not necessarily mislead the inferences of selective sweeps. Moreover, contrary to positive selection a reduction of the number of low frequency polymorphisms is be expected under background selection, generating positive values of Tajima’s D. In contrast, we found a consistent pattern of negative Tajima’s D across all the islands populations.

Lines 371-372: Though see Thompson et al. 2021. Patterns, Predictors, and Consequences of Dominance in Hybrids. American Naturalist.

**RESPONSE:** we have now included this reference and modified the sentence accordingly (see lines 973-974).

Line 376-378: “Indeed, we find that differentiation between the parent species explains neither the degree of differentiation within islands, nor improves the fit of the models evaluating within-island differentiation (logistic and GLM-models).” I find this sentence a bit confusing, because the authors predict a negative relationship (although see my above comments), but in this sentence seem to be citing a lack of relationship as evidence for this hypothesis.

**RESPONSE:** We agree and we have now modified the sentence accordingly (Lines 978-983).

Lines 422-423: “Taken together, our findings suggest that hybrid lineages may use their elevated levels of variation for local adaptation” I would avoid this anthropomorphic language.

**RESPONSE:** We agree and have now removed this sentence.

Line 425: “similar selection pressures” the authors haven’t really shown similar selective pressures (i.e. the agent of natural selection), but rather have shown that differentiation is correlated within and between islands, which may be a sign of parallel ecological adaptation, but also potentially background selection.

**RESPONSE:** We agree with the reviewer that we have not directly evaluated specific selective pressures, thus we have now modified this sentence to refer to the correlation of selection signatures within islands. We have also now included an analysis to differentiate between background selection and parallel adaptation, please see the response to the main question regarding background selection above (Fig. R9, Fig. R10 and Table R4).

Lines 448-450: “Furthermore, the island populations are evolutionarily independent and are hypothesized to have arisen from individual hybridization events.” Can the authors provide a reference for this?

**RESPONSE:** We have now added the correspondent reference and modified the sentence to clarify the evidence suggesting independent evolutionary origins on the islands’ populations (see Lines 1115-1121) in line with comments from reviewer #1. Please also see our response to point 2 from reviewer #1 above (Fig.R2).

**Reviewer #3:**

In this manuscript (“Predictors of genomic differentiation within a hybrid taxon”), the authors use a unique dataset of the replicated hybrid species the Italian sparrow to understand the consequences of hybridization at the genomic level. They discuss a number of clear hypotheses (Figure 1) outlining the potential roles of recombination rate, selection, and levels of differentiation (both within the hybrid species and between the parental species). I really enjoyed reading this manuscript and think it addresses a topic that is difficult to study in most vertebrate species! It really pushes our understanding of hybridization and genomics forward. The unique sampling approach—something that to my knowledge can’t be replicated in any other bird species—really makes answering their questions possible. I have a few major comments and a larger number of minor comments (sorry!) that I think will help make the manuscript more scientifically sound and easier to read, but overall, I think this is a really cool study and would fit really nicely in PLOS Genetics! (I also think the inclusion of hypothesis/prediction figures is really underdone in the genomics field, so it was really great to see such nice clear predictions presented! And the figures in general were very beautiful!)

**RESPONSE:** We are very happy that the reviewer enjoyed our manuscript and found that our study can push the boundaries to understanding hybridization processes. We thank the reviewer for the constructive comments helping us to improve the manuscript.

MAJOR COMMENTS:
In the introduction, I think you could more clearly separate the background information from the information introducing the Italian sparrow. On Line 100, you basically start discussing in-depth details about the sparrow system, but you’ve not yet officially mentioned that this will be the focus of the study. (There’s also a semi-extensive section from lines 58-62 about them, in a paragraph that’s more generally background information.) It’s a bit jarring going from a general discussion of the recombination rate hypothesis with Figure 1A, to the next paragraph describing the hypotheses for Figures 1B and 1C in the context of the Italian sparrow. I think you should either discuss all of the hypotheses in the context of the system, or all of them more generally. If you choose to do the former, then maybe moving the paragraph that starts on Line 122 up before you start to talk about any hypotheses would be useful. That way you’ve first introduced the system, and THEN go into details about hypotheses.

**RESPONSE:** We thank the reviewer for the comment and agree we should have introduced the study system much earlier. We have now modified the text to explicitly mention the Italian sparrow as our study system earlier in the introduction (Lines 76-83).

I think it would be great to explore the way the Crete population falls out in some of these clustering analyses. What happens if you run admixture or the PCA with just the two parental species and the Crete population? Do you get K=2 as the value with the highest support? Does Crete fall out between the two parentals in the PCA as you’d expect for a hybrid between the two? It just seems a little weird that both Corsica and Sicily have patterns more like what you’d expect for a hybrid species, but Crete is an oddball… You kind of touch on this briefly in the discussion and it seems like the explanation for the pattern is a genetically distinct house sparrow subspecies contributing to the Crete population? Given the patterns in Crete are pretty weird and it does sound like something different is going on there, are all three island populations you include really comparable? I don’t really see this as a huge issue, but it does seem like a bit more support that these are all comparable islands would be useful.

**RESPONSE:** The Crete population has previously been analyzed in Runemark et al 2018 *Nat Eco Evo* with a higher power analysis given the large number of loci from whole genome resequencing data. Runemark et al found a higher support for K=2 in an admixture analysis using the parental species, populations from the three islands evaluated in this study and an additional Mediterranean island (Malta). When performing a PCA with RAD data using only Crete and the two parental species the pattern is similar that the one presented in the original manuscript (see Figure R11)

**Figure R11.** PCA excluding two of the island populations, Sicily and Corsica, shows a similar pattern where Crete differentiate from the parental species in PC2, as previously shown in the original PCA (Fig. 2C) in the manuscript.

You are making so so many different comparisons throughout the paper with the added layer that many of these comparisons are of FST values (which is already a comparison). For the most part I was able to follow what comparisons you were making and why there were important, but I think it would be really helpful as you revise to make sure that you’re being really careful with the word choice you use to describe all the various different components and stick with them throughout. (island, parental, within, among, populations, minor parent, major parent… etc. etc.) I’ve tried to indicate in my specific comments below some places where things were a bit hard to follow.

**RESPONSE:** We thank the reviewer for this observation. We have now made sure the terms used are consistent throughout the manuscript. We also included a short explanatory sentence at the beginning of each results section to clarify what scientific question the comparisons are answering.

MINOR COMMENTS:
Line 4. It’s not clear what you mean by “contingencies” here

**RESPONSE:** We have now modified the manuscript gone over the terminology to ensure that concepts are not confounded including the term “contingency (e.g. Lines 4 and 128-131). Please also see the response to reviewer 2 on this topic.

Line 23. Typo in “a hybrid species result of”
**RESPONSE:** We have now modified this sentence.

Line 47. Citation 20 is a great reference for your work—and I think makes a lot of sense to include—but I’m not sure the current location/phrasing is the best way to do that. You just finish talking about hybrid species and hybrid speciation, and as swordtails are NOT an example of a hybrid species (more of a hybrid swarm), this might be confusing for readers.

**RESPONSE:** We changed the sentence to clarify that we are not referring to swordtails as hybrid species. This sentence refers to the dissimilar proportion of genetic parental contribution in hybrids and this is true in stabilized hybrid species as well as in hybrids from hybrid swarms like in the case of swordtails.

Line 72. Reduced introgression on the sex chromosomes is a pretty common pattern in birds, but the current phrasing makes it seem like the evidence is only present in a few cases…

**RESPONSE:** We agree and have now modified the sentence accordingly (Lines 131-147).

Line 81. I think this paragraph would be a great place to explicitly discuss Dobzhansky-Muller incompatibilities. They underly a lot of what you discuss in the intro, but you never explicitly describe them. (It would also really nicely flow into the ideas you discuss in the next paragraph!)

**RESPONSE:** We thank the reviewer for this helpful suggestion. We have now included the concept of BDMIs in the previous paragraph (113-118).

Line 130. What do you know about colonization of these islands? Did the hybrid species form first on the mainland and then subsequently colonize each island? Did the parental species colonize the islands and then separately hybridize? Do you have any information available on the timing of hybridization or colonization?

**RESPONSE:** Up to date we or other studies have not estimated the timing on these hybridization events. However, in Runemark et al., (2018) *Nature Ecol Evol*, the very low correlation of Spanish ancestry blocks found between pairwise comparisons of islands populations support a scenario where different populations of Spanish sparrows colonized the different islands independently. In line with comments from reviewer #1, please also see our response to point 2 from reviewer #1 (Fig. R2) above.

Line 150. Is there a specific place where you mention the ancestry proportions in each island population?

**RESPONSE:** We have modified the introduction and included the ancestry proportion of each island reported by Runemark et al., (2018). *Nat Ecol Evol.* (see Lines 79-83)

Line 176. “relation” should be “relationship”

**RESPONSE:** It’s now fixed

Line 195. Link back to your prediction figure here.

**RESPONSE:** Indeed. We have now included a sentence connecting this part of our analyses to the predictions made in the introduction (Lines 413-441).

Line 196. It would be helpful to explain what xp-EHH is in the text here.

**RESPONSE:** We agree and have included a short description of the statistic (Lines 440-441).

Line 198. Keep consistent how you refer to different island-island comparisons. Elsewhere you use a hyphen, but here you use “and.” There are just so many comparisons you’re making in this paper, it would be really useful if you were extra careful in being consistent with phrasing throughout!

**RESPONSE:** We thank the reviewer for this observation; we agree that being consistent with what terms are used will facilitate the understanding of the different analyses perform. We have now revised the manuscript for consistency.

Line 230. Is there a way to talk about this result that is more intuitive for the way the Figure 5D is presented? The figure has each island you’re comparing to grouped together on the x-axis, maybe you can switch the way you talk about the results to be similar.

**RESPONSE:** The paragraph is now modified to reflect the grouping of the results by island on the x-axis in Figure 5D.

Line 239. This section makes much more sense to me upon a re-read, but I did find it pretty confusing to wrap my head around on the first read.

**RESPONSE:** We thank the reviewer for pointing out how confusing this paragraph was. We have now rephrased it making it much simpler and hopefully easier for the reader to follow.

Line 251. Change “per island” to “for each island” ?

**RESPONSE:** This is now changed.

Line 252-3. You talk about Corsica and Sicily differently here: “between Corsican populations” and “within-Sicily differentiation” but the comparisons are the same I think.

**RESPONSE:** We have now consistently used the same terms throughout the manuscript.

Line 264. Is this result robust to more stringent cutoffs for FST outliers?

**RESPONSE:** While it would be interesting to look into, we think that the manuscript already includes quite many analyses and therefore prefer to present one cutoff only.

Line 265-273. There have been a few points throughout this section where it would be helpful to have an explicit statement about who the minor parent is for each island (e.g., Line 259, 264). I think it would be really helpful to have this info presented much earlier. I found this part of the results to be pretty confusing—I think because there are a lot of comparisons being made, but the expectations for minor/major parent ancestry haven’t really been well-established or discussed yet. I see similar patterns across all of the islands in Figure 6B, but I don’t think that is your expectation and I’m having a hard time squaring the results as I interpret them in the figure and this part of the results.

**RESPONSE:** We have now mentioned the minor-parent for each island at the beginning of this same paragraph (Lines 621-623) and early in the introduction we provided the estimated admixture proportions (Lines 79-83). We have also modified this paragraph to clarify the expectations of minor- major- ancestry and the results presented in Fig. 6B

Line 287. More explicitly introduce the hybrid index here?

**RESPONSE:** We have now included a sentence introducing the hybrid index, now called per-locus-local-ancestry-proportion (LLAP), all to clarify the nature of this estimate.

Line 306. Typo “in the light of” should be “in light of”

**RESPONSE:** This is now fixed.

Line 307. It might be helpful to state how dispersal distance compares to island size?

**RESPONSE:** We have now mentioned that the high genomic differentiation among populations within islands found is present at relatively short distances between each other in light of the dispersal ability of the species according to Summers-Smith, J. D. (1988).

Line 315. Tie back to your prediction figures?

**RESPONSE:** We thank the reviewer for the comment and have now included a sentence linking these conclusions to our initial predictions formulated in Fig. 1.

Line 317. Should “island” be plural here?

**RESPONSE:** Indeed. We have now changed this sentence.

Line 354. There are other bird examples too, the current phrasing makes it sound like the evidence is kind of limited. E.g., Van Doren et al. 2017 shows a similar pattern, and I think so does some of Darren Irwin’s papers on warblers.

**RESPONSE:** We agree and have now included the new references the reviewer suggested (Lines 931).

Line 405. Typo “affect the effect”

**RESPONSE:** We have now modified this sentence.

Line 437. Maybe use “minor parent ancestry” here as you have elsewhere?

**RESPONSE:** As we performed a new analysis tackling the issue mentioned here, this sentence has now changed.

Line 450. This seems worthwhile to mention sooner!

**RESPONSE:** We included more explicit information regarding results that suggest independent hybrid events in this sentence (Lines 1115-1121) and mentioned it earlier in the introduction (see Lines Lines 254-258).

Line 465. It would be useful to indicate that you used a RADseq approach in the Results section.

**RESPONSE:** We now included this information along the results (e.g. see Line 325) and in the figures legend (see Fig.2, Fig.3, Fig.4).

Line 468. What length of reads did you sequence? On what platform?

**RESPONSE:** This information is now included.

Line 476. Typo “re-aligned indels realignment”

**RESPONSE:** Revised. Thanks for bringing this to our attention.

Line 482. Add the number of SNPs here.

**RESPONSE:** As we used different VCF files containing different set of individuals and SNPs we have provided this information in the supplementary table S2. However, we now also included this information in the description and figure legend for the most relevant analyses. However, we believe that specifying the number of SNPs for all different VCFs in the main text would not improve the reading experience.

Line 487. Weird capitalization in PCA

**RESPONSE:** Agreed. This is now fixed.

Line 493-494. Is this sentence out of place?

**RESPONSE:** This section is now revised.

Line 496. Was this also in `adegenet`?

**RESPONSE:** It was indeed, and this is now specified in the text.

Line 517. This is just one sentence in the supplement, right? If so, just move to the main text.

**RESPONSE:** We thank the reviewer for the suggestion. We have now moved that sentence to the manuscript.

Line 540-542. Move this information to the Results when you talk about xp-EHH

**RESPONSE:** We have now also specified xp-EHH statistic (see Lines 439-441).

Line 565. As I mentioned earlier, I think it would be great to investigate if more stringer outlier cutoffs lead to similar results or not.

**RESPONSE:** While we agree it would be interesting, this is a complex read with many results as it is, and we do not think including several cut-offs would change the results in any substantial way or improve the reading experience. We could, however, include additional cut-offs in a future revision if the editor recommends so.

Line 572-576. The info you include here on the GLM might be helpful in interpreting those analyses in the Results; maybe move earlier?

**RESPONSE:** We have now moved that information to the results section (see Lines 583-587).

Line 579. Typo “locus to belong to the” should be “locus belonging to the”

**RESPONSE:** Indeed. We have now revised the sentence accordingly.

Line 591-592. I think there’s an error in the description of major vs. minor parent ancestry?

**RESPONSE:** We have now revised the wording to make the description of the analysis more straight forwards. We selected genomic blocks where frequencies of parental alleles are high, i.e. genomic blocks with frequencies of >65% of major-parent alleles and >65% of minor-parent alleles. In such extremes ranges of the distribution we evaluated whether those loci involved in within-island differentiation are disproportionally more often of minor parent ancestry (>65% of minor-parent alleles) than what be expected by chance.

Line 593. How does the choice of just 8 outlier loci influence the results? How does choosing different numbers influence things?

**RESPONSE:** This is a very conservative approach, as 8 is the lowest number of loci of the minor-parent in the outliers set present across the islands.

Line 595. It might be helpful to bring up this information on how the HI is calculated to have it be the second sentence in this paragraph.

**RESPONSE:** We agree and thank the reviewer for this suggestion. This paragraph is now presented at an earlier stage.

COMMENTS ON FIGURES/TABLES:
Figure 1. maybe adding arrows to panel A would help with matching to your description in the legend? More generally, make sure to link back to panels B and C when you’re discussing those hypotheses in the results section.

**RESPONSE:** We have now substantially modified figure 1, in particular the legend, to facilitate interpretation. We also ensured to refer back to the hypotheses proposed here in the results and discussion.

Figure 3. (A) the y-axis on this plot is pretty confusing…

**RESPONSE:** We thank the reviewer for bringing our attention to this figure. We agree and have now included a description of the y-axis in the legend to clarify the dashed line.

Figure 4. (A) typo in panel legend (“v.s” should be “vs.”) and in a number of the supplemental tables); (D) would it be useful to include a line in this plot, given the significant correlation?

**RESPONSE:** We agree and have now modified the figure accordingly. We thank the reviewer for this great suggestion.

Figure 5. (A) make sure p-values match exactly what you include in the text for ease of identification of the exact test; maybe add lines to indicate the significant correlations in the two comparisons here? (B) add lines here too to show the significant correlations? (C) left/right indications are switched in the legend

**RESPONSE:** We thank for bringing our attention to these issues. The figure now has been fixed.

Figure 6. (A) You use the black versus colored points in this figure and in a number of figures in the supplement, but the only place you really mention what the difference between the two points means is in Figure S7. I assume it means the same thing throughout other figures (outlier vs non-outlier SNPs), but it would be nice to have that indicated somewhere in this figure (as it’s the only in-text one). And again, might be useful to add lines to indicate the significant correlations.

**RESPONSE:** The distinction between colored dots as 1% FST outliers and non-outliers in black is now mentioned in the legend of this figure, and all other figures where this distinction is valid. Thanks for bringing this to our attention!

Table 1. Several typos in table legend (“were use” should be “were used”; “did not chance” should be “did not change”; “was maintained” should be “were maintained”)

**RESPONSE:** We thank the reviewer for spotting so many typos! This has now been changed.

Figure S1. Could probably remove if you wanted, I’m not sure it adds much to the paper (given the results are shown in a table).

**RESPONSE:** We thank the reviewer for the suggestion.

Figure S2. Same comment as for Figure S1.

**RESPONSE:** We thank the reviewer for the suggestion.

Table S3. It took me a little while to really understand what was going on within each section of this table. I understand not wanting to split it into three separate tables, but some additional guidance on how to read the info in the table might be helpful!

**RESPONSE:** We have included more information on the table legend to clarify the interpretation of statistics presented here.

Table S6 and S7. Switch the order? You mention S7 first in the text (Line 188), so maybe make it S6.

**RESPONSE:** The order of the tables has now been changed.

Table S8. Maybe change “per island” to “separated by island” in the title?

**RESPONSE:** We have now changed it.

Table S9. Missing closing parentheses

**RESPONSE:** Indeed. This is now fixed.
