# Supplementary material for: Predictors of genomic differentiation within a hybrid taxon
Source: PLoS Genet. 2022 Feb 11;18(2):e1010027. doi: 10.1371/journal.pgen.1010027 (PMC8870489; doi:10.1371/journal.pgen.1010027)
Supplement: Attachment — Submitted filename: Response.Letter_Cuevas.etal.2021_PLoSGenetics_MinorR_F.docx [file pgen.1010027.s006.docx]

**January 2^nd^ 2022**

Dear Editor,

We are happy to resubmit our manuscript entitled “Predictors of genomic differentiation within a hybrid taxon” for consideration following a minor revision. This is a revised version of our manuscript PGENETICS-D-21-00438. We have carefully addressed all suggestions and comments made by the Editor and reviewers. In particular we have made the following changes:

- Included test on recombination rate and minor-parental ancestry in the supplementary material.
- Modified figure 1A following the recommendations of reviewer # 2 to further clarify the expectations for the patterns of differentiation.
- Corrected the order of the references
- Carefully checked for grammatical errors and typos

We have carefully revised the manuscript, taking all suggestions by the reviewers into consideration. Please find a detailed response to the editor and reviewer’s comments below. We hope the editor will find this revised version suitable for publication in PLoS genetics. We anticipate this research will provide crucial new insights into the evolutionary processes molding hybrid genomes, with implication for the potential of hybridization to contribute new diversity.

Sincerely yours,

**Angélica Cuevas (corresponding author)**

[a.m.c.pulido@ibv.uio.no](mailto:a.m.c.pulido@ibv.uio.no), [angelica.cuevas@evobio.eu](mailto:angelica.cuevas@evobio.eu)

Centre for Ecological and Evolutionary Synthesis

University of Oslo, Norway

<https://www.mn.uio.no/cees/english/people/phd/angelimc/>

**Comments to the Authors:**

This revised manuscript has been evaluated by two of the original referees. I apologize for the delay as we sought to also obtain a review from the third original referee. The two reports express their appreciation for the revisions to the manuscript and thorough responses to the previous round of review. I concur. One review notes a few copy-editing points related to clarity that I encourage the authors to address. It would be a good idea at this stage for the authors to work through the copy carefully to find any additional usage and typographical errors. Otherwise I believe the authors have done a very thorough job of responding to review and that this manuscript will be a good addition to our growing understanding of the composition of hybrid lineages.

**RESPONSE:** We are very happy that the editor finds our revisions to be satisfactory, and thinks that our study will be a good contribution to the field. We have carefully addressed the copy-editing points raised, and checked the manuscript for additional typos.

**Reviewer's Responses to Questions**

**Reviewer #2:**

In their manuscript entitled ‘Predictors of genomic differentiation within a hybrid taxon’ Cuevas et al. sequence a series of populations on three islands of a homoploid hybrid species, the Italian Sparrow, and use various population genomics methods to compare differentiation within and between islands, as well as between hybrids and the two progenitor species to better understand what factors influence the resolution of hybrid genomes. Specifically, they aim to understand whether differentiation (measured by Fst) within hybrids is a factor of recombination, selection, or the major/minor ancestry of that hybrid population. I think this work is both timely and very important for our field, and I agree that the authors have a powerful system to address these questions.

This is the second time that I have reviewed this manuscript, and I applaud the authors for submitting such a thorough revision! I have only a few residual major comments.

**RESPONSE:** We are very happy that the reviewer thinks the work is timely and important for the field, and that we were able to address most points in our previous round of revision.

**First**, I think the figure displaying proportion minor ancestry vs recombination bins would be very useful in the supplement.

**RESPONSE:** We thank the reviewer for this suggestion. We have now included a figure displaying proportion of minor parent ancestry divided per recombination bin in the Supplementary Material (Fig. S13, Table S14). We have also discussed these patterns in the manuscript (Lines 400 – 407; 515 - 517)

**Second**, in Fig 1 A, given that the variation between hybrid populations is merely a subset of variation between parents, it seems like the hybrid-hybrid differentiation should always be lower than differentiation between parents across all recombination rates (incidentally, this is also what the authors find). I would recommend making this minor change to the figure.

**RESPONSE:** Initially we were focused on the differences on the slope of the lines as we presented in the figure caption and in the main text of the manuscript, but neglected the difference in the level of genomic differentiation that can occur when comparing hybrid populations among themselves in comparison to that between parental species. While there is a theoretical possibility that one hybrid lineage fixes one parental allele and the other the alternate parental allele, this is not expected to be common. Thus, as the reviewer suggested the hybrid-hybrid comparison is expected to present lower genomic differentiation than that between parental species. We thank the reviewer for this observation and have now modified the Fig. 1A accordingly.

**Third**, I think the references have been shifted- for example, line 494 uses a cichlid reference for warblers, and the reference for dominance and novel trait expression is listed as Andrews (2010) (the reference for FastQC), rather than the appropriate Thompson et al. 2021 (which is listed as two before in the reference list.

**RESPONSE:** We have now corrected the numbering of the references. We are thankful with the reviewer for spotting this mistake.

Minor comments:

There are also quite a few grammatical errors and typos:

**RESPONSE:** We thank the reviewer for carefully going over the manuscript and identifying our errors and typos. We have now corrected them.

Line 12: should “signature” be “signatures”?

**RESPONSE:** We have corrected this error now.

Line 84-86: This sentence is strangely structured- the topic of the sentence is the variation between taxa, but then the example is how loci can vary. I would rephrase to just say something like “The speed of genomic stabilization varies between hybrid taxa and among loci…” or something.

**RESPONSE:** This sentence has now been modified.

There are several instance of extraneous ‘e.g.’ and ‘i.e.’s that I think add to confusion rather than clarify the writing (for example, lines 89, 101, 256, 440, 492, 536,).

**RESPONSE:** We have now omitted these abbreviations to make the reading easier.

Line 300: should “island” be “islands”?

**RESPONSE:** This typo has been now corrected.

Line 343: first mention of how Fst outliers are defined (top 1%), but first mention of outliers occurs ~line 234. Could the authors please define outliers the first time they’re mentioned?

**RESPONSE:** We have now defined F_ST_ outliers in line 234. We thank the reviewer for spotting this mistake.

Line 359-60: “While Crete and Sicily present a higher ratio of fixed loci from the house and Spanish sparrow, respectively” is not a grammatically correct sentence.

**RESPONSE:** We have now modified this sentence.

Line 441: Should “vas” be “vast”?

**RESPONSE:** This typo has been corrected.

Line 512: this would only be true in regions of low divergence AND low within species diversity…

**RESPONSE:** We have now modified the sentence.

Line 598: Should this read “across the same windows”?

**RESPONSE:** Indeed. We have now corrected this sentence.

Lines 671-73: Where are these results presented?

**RESPONSE:** We have now included them in the supplementary material (Table S15).

**Reviewer #3:**

I commend the authors on the revisions to this manuscript—it’s much improved! I am satisfied with how the authors addressed my comments and the comments from the other reviewers and editor. I particularly appreciated the revisions to the introduction/framing of the paper and the additional analytical checks to confirm the patterns that are being presented. I think the study is really interesting (!) and the unique biology/sampling of the Italian Sparrow system will be of broad interest to the readership of PLOS Genetics.

**RESPONSE:** We are very happy that the reviewer found our revision satisfactory. We thank the reviewer for providing the comments enabling us to improve on the manuscript, and we are happy that the reviewer thinks this study will be of broad interest to the readership.
